# Efficacy and Safety of Moxidectin, Synriam, Synriam-Praziquantel *versus* Praziquantel against *Schistosoma haematobium* and *S*. *mansoni* Infections: A Randomized, Exploratory Phase 2 Trial

**DOI:** 10.1371/journal.pntd.0005008

**Published:** 2016-09-16

**Authors:** Beatrice Barda, Jean T. Coulibaly, Maxim Puchkov, Jörg Huwyler, Jan Hattendorf, Jennifer Keiser

**Affiliations:** 1 Department of Medical Parasitology and Infection Biology, Swiss Tropical and Public Health Institute, Basel, Switzerland; 2 University of Basel, Basel, Switzerland; 3 Unite´ de Formation et de Recherche Biosciences, Universite´ Felix Houphouët-Boigny, Abidjan, Côte d’Ivoire; 4 Centre Suisse de Recherches Scientifiques en Côte d’Ivoire, Abidjan, Côte d’Ivoire; 5 Department of Pharmaceutical Technology, University of Basel, Basel, Switzerland; 6 Department of Epidemiology and Public Health, Swiss Tropical and Public Health Institute, Basel, Switzerland; Ohio State University, UNITED STATES

## Abstract

**Background:**

Schistosomiasis affects millions of people, yet treatment options are limited. The antimalarial Synriam (piperaquine 150 mg/arterolane 750 mg) and the anthelminthic moxidectin revealed promising antischistosomal properties in preclinical or clinical studies.

**Methodology:**

We conducted two single-blind, randomized exploratory Phase 2 trials in *Schistosoma mansoni* and *S*. *haematobium*-infected adolescents in northern and central Côte d’Ivoire. Our primary endpoints were cure rates (CRs) and egg reduction rates (ERRs) based on geometric mean and safety. Each subject was asked to provide two stool samples (*S*. *mansoni* trial) for Kato-Katz analysis or three urine samples (*S*. *haematobium* trial) for urine filtration and one finger prick for malaria screening at baseline and follow-up. Participants were randomly assigned to either moxidectin, Synriam, Synriam plus praziquantel or praziquantel.

**Principal Findings:**

128 adolescents (age: 12–17 years) were included in each study. Against *S*. *haematobium* moxidectin and Synriam revealed low efficacy. On the other hand, Synriam plus praziquantel and praziquantel yielded CRs of 60.0% and 38.5% and ERRs of 96.0% and 93.5%, respectively. CRs observed in the treatment of *S*. *mansoni* were 13.0%, 6.7%, 27.0%, and 27.6% for moxidectin, Synriam, Synriam plus praziquantel and praziquantel, respectively. ERRs ranged from 64.9% (Synriam) to 87.5% (praziquantel).

**Conclusion/Significance:**

Synriam and moxidectin show low efficacy against *S*. *haematobium*, hence an ancillary benefit is not expected when these drugs are used for treating onchocerciasis and malaria in co-endemic settings. Further studies are needed to corroborate our findings that moxidectin and Synriam show moderate ERRs against *S*. *mansoni*.

## Author Summary

Schistosomiasis is a parasitic infection that affects millions of people all over the world and it is due to schistosomes, helminths (worms) that infect the intestine and the urinary bladder. Treatment options are limited, with praziquantel being the only used drug. The antimalarial Synriam and the anthelminthic moxidectin revealed good action against this worm in previous studies. We conducted two studies in *Schistosoma mansoni* and *S*. *haematobium*-infected adolescents in Côte d’Ivoire. Subjects positive for the infection were allocated by chance to the four groups of treatment (moxidectin, Synriam, Synriam plus praziquantel or praziquantel); participants did not know which drug they took. Our aim was to calculate how many participants were negative after the treatment and how did the intensity of infection change before and after treatment. Each subject provided stools and urines for examination. 128 adolescents were included in each study. Moxidectin and Synriam did not work well against *S*. *haematobium*. Against *S*. *mansoni*, only a small part of the participants were negative after treatment in all treatment groups, but the intensity of infections were reduced. Further studies are needed to better understand this result.

## Introduction

Schistosomiasis caused by the three main species *Schistosoma haematobium*, *S*. *japonicum* and *S*. *mansoni* is a disease known since ancient times. An estimated 230 million people are infected and the disease causes a burden of 3.0 million disability-adjusted life years lost [1, 2]. Despite the enormous public health problem with regard to symptomatology and morbidity, it is listed among the so-called neglected tropical diseases (NTDs) [3]. The treatment of schistosomiasis relies on one drug only, praziquantel, which is active against adult schistosomes, but has little activity against juvenile worms [3]. The high drug pressure resulting from the widespread administration of praziquantel in the framework of preventive chemotherapy programs could lead to drug resistance [4]. However, at the moment there are no viable alternatives to praziquantel [5]. Even so, drug discovery has languished, and no drug is currently undergoing clinical testing [6]. Therefore, the discovery and development of new drugs for the treatment of schistosomiasis is a high priority.

The antischistosomal activity of the artemisinins was described more than 30 years ago [7]. Subsequently, many studies have been conducted to elucidate the antischistosomal effect of the artemisinins and synthetic peroxides [8, 9]. The 1,2,4-trioxolanes in particular, characterized by improved pharmacokinetic parameters compared to the artemisinins [10], were the focus of different *in vitro* and *in vivo* studies, which demonstrated efficacy not only against *Schistosoma* spp. but also against *Echinostoma caproni*, *Fasciola hepatica* and *Clonorchis sinensis* [9, 11]. In more detail, OZ78, OZ277 and OZ209 were first studied in *S*. *mansoni* and *S*. *japonicum* rodent models more than a decade ago. A particularly high activity of the 1,2,4-trioxolanes was observed against juvenile *S*. *mansoni* and *S*. *japonicum* infections in the mouse model and against both juvenile and adult stages of the worms in the hamster model [11]. After licensing OZ277 (arterolane maleate) in combination with piperaquine in 2011 (Synriam) [12–14] Mossallam and colleagues studied the efficacy of OZ277 in combination with piperaquine in rodents infected with *S*. *mansoni*, which confirmed the excellent activity of this trioxolane derivative particularly against the worms’ juvenile stages [15].

Moxidectin is widely used in veterinary medicine for heartworms [16]. Studies are ongoing to develop moxidectin as potential alternative to ivermectin for the treatment of onchocerciasis [17]. In the framework of this drug development program effects of moxidectin on concomitant helminths were studied [18], revealing cure rates (CR) and egg reduction rates (ERR) of 64% and 66%, respectively in *S*. *mansoni*-infected patients [19].

We conducted two single-blinded, randomized exploratory Phase 2 trials in *S*. *haematobium* and *S*. *mansoni-*infected adolescents to assess the efficacy of moxidectin and Synriam. One group of children was treated with a combination of Synriam and praziquantel since this combination might offer effects against pre-patent and patent infections. Finally, praziquantel treated participants served as active control.

## Methods

### Ethics statement

Ethical clearance was obtained from the ethics committee of Northern and Central Switzerland (EKNZ; reference no. 15/01) and from the Comité National d’Éthique et de la Recherche du Ministère de la Santé et de l’Hygiène Publique (reference no. 026) in Côte d’Ivoire. The trial is registered with Current Controlled Trials (ISRCTN 63657086). Participants aged 12–18 years old were eligible for inclusion in the trial. Written informed consent was obtained before enrolment by the children aged 18 years and by parents or legal guardians of the children below 18 years old. The latter assented orally.

### Study setting and population

The single-blind, randomized, exploratory, four arm Phase 2 trials were conducted in May and June 2015, in the health districts of Toumodi (Moronou village (geographical co-ordinates 06°19’0” N latitude, 04°58’0” W longitude), endemic for *S*. *haematobium*) and Man (villages of Bigouin (7°24’01” N, 7°33’11” W) and Biakalé (7°27’07” N, 7°41’32”), endemic for *S*. *mansoni*) of Côte d’Ivoire. In all locations, village based recruitment was implemented.

### Randomization and drugs

We used a computer-generated block randomization code stratified by baseline infection intensities (block size of 8) provided by an independent statistician. Enrolled subjects were randomly allocated to the four treatment arms i) single dose of moxidectin liquid formulation 8 ml (8 mg), ii) Synriam (150 mg arterolane plus 750 piperaquine): three doses administered for three consecutive days, iii) Praziquantel 40 mg/kg single dose plus Synriam (150 mg arterolane plus 750 piperaquine) three doses administered for three consecutive days, iv) Praziquantel 40 mg/kg single dose. Since drug interactions were not yet studied, the combination treatment group received praziquantel in the morning followed by Synriam in the late afternoon (and two additional Synriam courses over the next two days). Only the study investigator was aware of the treatment assignments, while children and laboratory technicians were blinded. Moxidectin was administered as oral suspension (Cydectin 0.1% Zoetis, Switzerland) mixed with equal amounts of mint syrup (sweetener RK50 (E952 (Sodium Cyclamate), E954 (Sodium Saccharin); Peppermint Plus Aroma and Citrus Plus Aroma [Rohner Konzept, Switzerland]) to mask the bitter taste of the drug. Praziquantel was administered based on subjects’ weight using 600 mg tablets (Cesol, Merck).

### Study procedures

Community meetings were conducted to explain the purpose, procedures, potential risks and benefits of the study. At baseline three urine samples (*S*. *haematobium* cohort) and two stool samples (*S*. *mansoni* cohort) were collected from participants. Schistosome-positive adolescents, who had provided 2 stool and 3 urine samples were eligible to participate in the trials. One stool sample was collected from each participant in the *S*. *haematobium* trial and one urine sample from each patient participating in the *S*. *mansoni* trial to evaluate co-infections.

The standard urine filtration method (10 ml of urine) was used for appraisal of *S*. *haematobium* infections [3, 20]. Microhematuria was assessed on one urine sample using Hemastix (Siemens, Munich, Germany) dipsticks. The Kato-Katz technique was used for the quantitative assessment of *S*. *mansoni* infections. Duplicate Kato-Katz was performed on each stool sample using the 41.7 mg template according to the standardized method [21]. All slides were double-checked by a second laboratory technician; slides were considered negative only if no parasites were detected by the two independent microscopists. Concomitant infections with soil-transmitted helminth (STH) infections were recorded. In addition, we used the circulating cationic antigen (CCA) tests (ICT diagnostics, Cape Town, South Africa) on one urine sample for *S*. *mansoni* diagnosis. The POC-CCA cassette (batch 33112) was performed according to the manufacturer’s instructions. The test results were scored as negative or positive, the latter stratified into trace (very light color band), 1+, 2+ (light infection), and 3+ (heavy infection) according to the visibility of the color reaction. On the day of treatment and physical examination, participants provided one finger prick sample for malaria and hemoglobin testing. The hemoglobin concentration was determined using a portable Hemocue 301 (HemoCue AB; Ängelholm, Sweden). Additionally, a rapid malaria diagnostic test (RDT) (ICT diagnostics) was employed. Thick and thin blood smears were prepared on a microscope slide for subsequent appraisal of malaria parasitemia. The medical history of participants was assessed with a standardized questionnaire, in addition to a clinical examination carried out by the study clinician. Height was measured with a standard meter (to the nearest 1 cm) and weight with an accurate electronic balance (to the nearest 0.1 kg). After treatment adverse events were monitored at 3, 24, 48 and 72 hours after each dose of treatment was administered. Participants were excluded if they suffered from any systematic illness (e.g. clinical malaria).

At day 21 after the last treatment dose was provided we sampled again 3 urine and 2 stool specimens for analysis of *S*. *haematobium*, *S*. *mansoni* and STH infections, together with a finger prick for the diagnosis of malaria infection. At the end of the study all participants positive for *S*. *mansoni*, *S*. *haematobium*, and STH infections were treated with albendazole (400 mg) and/or praziquantel (40 mg/kg) and artesunate and lumefantrine following national guidelines for malaria treatment.

### Sample size and statistical analysis

We aimed for 25 participants per treatment arm, a common sample size for exploratory Phase 2 trials [22]. Allowing for an attrition rate of up to 20%, it was planned to include 30 participants per treatment arm. For the estimation of the prevalence of *S*. *mansoni* and *S*. *haematobium* infection in these settings we based our calculation on a previously reported prevalence of 60–70% at nearby sites [23, 24]. However, we used a conservative estimate of 60% for our sample size determination and hence we planned to screen 200 subjects in each study cohort to detect at least 120 eligible participants infected with *S*. *mansoni/S*. *haematobium*.

Results were double entered in a database (Excel 2010), cross-checked and analyzed with Stata 12.0 (Lakeway Drive College station, TX, Unites States of America). An available case analysis was performed, which included all children with primary outcome data. The intensity of *S*. *mansoni* infection (number of eggs per gram (epg) of feces) was assessed by adding up the egg counts from the quadruplicate Kato-Katz thick smears (from baseline and follow-up separately) and multiplying this number by a factor of six [25]. The intensity of infection for *S*. *haematobium* was assessed by calculating the average of the egg counts from the triplicate urine filtration. Infection intensity was classified following WHO cutoffs [26]. Geometric and arithmetic-mean egg counts were calculated for each group before and after treatment. Egg reduction rates (ERRs) were calculated by the following formula (ERR = (1-(geometric mean at follow-up/geometric mean at baseline))*100). Bootstrap resampling method with 2,000 replicates were used to calculate 95% confidence intervals (CIs) for ERRs. Differences in ERRs were determined under the assumption that non-overlapping CIs indicate statistical significance. Cure rates (CRs) were calculated as the percentage of children who became egg-negative after treatment, being egg-positive at baseline.

## Results

### Study flow

**Fig 1:**
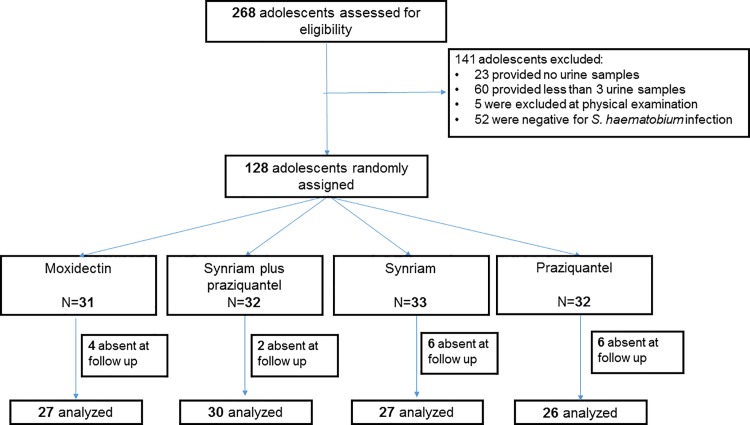
Study flow in the *S*. *haematobium* study.

**Fig 2:**
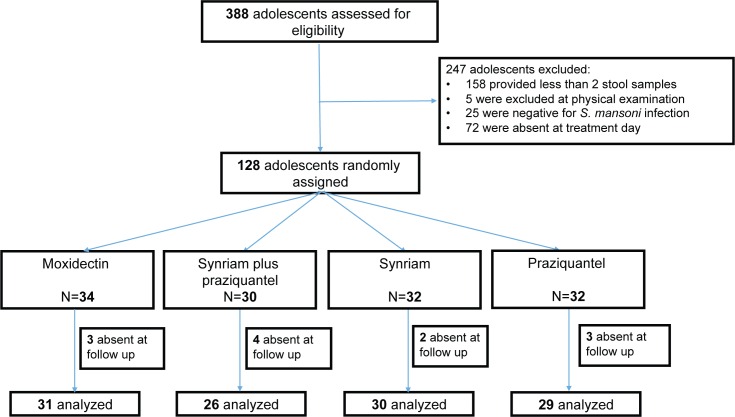
Study flow in the *S*. *mansoni* study.

The study flowcharts are presented in Figs 1 and 2. In the *S*. *haematobium* study we screened 268 children/adolescents of which 52 were negative for infection, 23 did not provide any urine sample, 60 delivered less than 3 urine samples, and 5 were excluded at physical examination because they did not meet the inclusion criteria. In total 128 participants were enrolled and randomly assigned to one of the four treatments as follows: 31 subjects received moxidectin, 32 were treated with Synriam plus praziquantel, 33 were administered Synriam and 32 were treated with praziquantel. Of all participants, 110 were present at the follow up examination and 18 were lost to follow up (Fig 1).

In the *S*. *mansoni* study we screened 388 subjects among whom 25 were negative for a *S*. *mansoni* infection, 158 did not provide 2 stool samples, and 72 did not appear at the treatment venue. Five subjects were excluded because they did not meet the inclusion criteria.

Participants were randomly assigned to either moxidectin (n = 34), Synriam plus praziquantel (n = 30), Synriam (n = 32) and praziquantel (n = 32). In total we treated 128 subjects and 116 were present at the follow up examination (12 lost to follow up) (Fig 2).

### Baseline characteristics

Demographic and clinical baseline characteristics are summarized in Tables 1 and 2. Treatment groups in the *S*. *haematobium* trial were well balanced in terms of age (mean age: 13.5 years, range: 12–17 years), sex (58.6% male participants), weight (mean weight: 39.4 kg), height (mean height: 149.2 cm) and baseline infection intensity. The arithmetic and geometric mean number of *S*. *haematobium* eggs per 10 ml was 53.4 and 17.1, respectively. 70% of participants had light and 30% moderate to heavy infections. Twenty-eight (21.9%) of the 128 treated subjects had a coinfection with STH. Microhematuria was detected in 61 (49%) subjects. Eighty-two (65.6%) of the 128 treated subjects were malaria positive based on thick and thin smears and 72.6% based on RDT.

**Table 1:** Baseline characteristics of adolescents infected with *S*. *haematobium* stratified by treatment group. The study was carried out in the villages of Moronou in central Côte d’Ivoire, between May and June 2015.

	Moxidectin (N = 31)	Synriam plus praziquantel (N = 32)	Synriam (N = 33)	Praziquantel (N = 32)	Total (N = 128)
Age (years) [mean (SD)]	13.8 (0.25)	13.6 (0.2)	13.5 (0.2)	13.3 (0.2)	13.5 (1.1)
Males N (%)	20 (64.5)	19 (59.4)	20 (60.6)	16 (50)	75 (58.6)
Hemoglobin (g/dl) [mean (SD)]	11.9 (0.1)	11.9 (0.2)	11.7 (0.1)	11.8 (0.2)	11.9 (0.1)
Weight (kg) [mean (SD)]	41.0 (1.6)	38.8 (1.3)	38.0 (1.8)	39.9 (1.9)	39.4 (0.8)
Height (cm) [mean (SD)]	150.0 (2.0)	148.8 (1.5)	148.9 (1.9)	148.9 (1.9)	149.2 (0.9)
EPG (AM)	55.0	52.4	53.9	52.1	53.4
EPG (GM)	16.8	16.6	16.9	18.2	17.1
Infection intensity N (%)					
Light	22 (71.0)	23 (72.0)	23 (70.0)	22 (69.0)	90 (70.3)
High	9 (29.0)	9 (28.0)	10 (30.0)	10 (31.0)	38 (29.7)
Microhematuria positive N (%)	17 (57.0)	14 (46.0)	16 (59.0)	14 (44.0)	61 (49.0)
STH coinfection N (%)	5 (16.1)	7 (21.9)	9 (27.3)	7 (21.9)	28 (21.9)
Malaria RDT positive N (%)	20 (64.5)	22 (68.7)	26 (78.7)	25 (78.1)	93 (72.6)
Malaria direct smear positive N (%)	20 (64.5)	22 (68.7)	18 (58.0)	22 (71.0)	82 (65.6)

SD, standard deviation; AM, arithmetic mean; GM, geometric mean, EPG, eggs per gram, RDT, rapid diagnostic test

**Table 2:** Baseline characteristics of adolescents infected with *S*. *mansoni* stratified by treatment group. The study was carried out in the villages of Bigouin and Biakalé in western Côte d’Ivoire, between May and June 2015.

	Moxidectin (N = 34)	Synriam plus praziquantel (N = 30)	Synriam (N = 32)	Praziquantel (N = 32)	Total (N = 128)
Age (years)	12.9 (0.2)	12.7 (0.2)	12.8 (0.3)	12.8 (0.3)	12.8 (0.1)
Males N (%)	17 (50.0)	22 (73.3)	15 (46.8)	17 (53.1)	71 (55.5)
Hemoglobin (g/dl) [mean (SD)]	12.2 (0.1)	11.7 (0.2)	11.7 (0.2)	11.8 (0.2)	11.8 (0.1)
Weight (kg) [mean (SD)]	34.4 (1.5)	32.8 (1.5)	39.8 (2.2)	33.97 (1.2)	35.2 (0.8)
Height (cm) [mean (SD)]	138.5 (2.0)	136.2 (2.5)	144.7 (2.1)	137.8 (1.8)	139.3 (1.1)
EPG AM	207.5	249.8	191.7	219.8	216.6
EPG GM	100.9	103.2	99.2	139.9	109.1
Light intensity N (%)	17 (50)	13 (43.3)	15 (46.8)	14 (43.8)	59 (46.1)
Moderate intensity N (%)	13 (38.2)	11 (36.7)	13 (40.6)	12 (37.5)	49 (38.3)
High intensity N (%)	4 (11.8)	6 (20.0)	4 (12.5)	6 (18.7)	20 (15.6)
CCA N (%)	29 (90.6)	27 (90.0)	26 (86.7)	29 (90.0)	111 (89.5)
Trace N (%)	2 (6.3)	4 (13.3)	4 (6.7)	0 (0)	8 (6.5)
Low intensity N (%)	5 (15.6)	2 (6.7)	4 (13.3)	3 (3.4)	14 (11.3)
High intensity N (%)	22 (68.8)	21 (70)	20 (66.7)	26 (81.3)	89 (71.8)
STH coinfection N (%)	12 (35.3)	16 (53.3)	13 (40.6)	8 (25.0)	49 (38.3)
Malaria RDT positive N (%)	24 (70.6)	24 (80)	26 (81.2)	25 (78.1)	99 (77.3)
Malaria direct smear positive N (%)	28 (82.3)	22 (73.3)	24 (75)	27 (84.4)	101 (78.1)

SD, standard deviation; AM, arithmetic mean; GM, geometric mean, EPG, eggs per gram, RDT, rapid diagnostic test

Among the participants in the *S*. *mansoni* trial, 55.5% were male and the mean age was 12.8 (12–17) years (Table 2). No differences among treatment arms were observed in terms of weight (mean weight 35.2 kg), height (139 cm), gender (71 males) except from the Synriam plus praziquantel treatment arm, in which 73% of participants were males. The arithmetic and geometric mean *S*. *mansoni* infection load was 216.6 and 109.1 epg, respectively. 59 (46.1%) of adolescents had light infections and 69 (53.9%) moderate to heavy infections. 49 (38.3%) of participants had a coinfection with STH. 101 (78.1%) of the *S*. *mansoni*-infected adolescents were malaria positive according to thick and thin smear and 99 (77.3%) according to the RDT.

### Efficacy against *S*. *haematobium* and *S*. *mansoni*

We observed a low efficacy for the two novel treatments in the *S*. *haematobium* cohort (Table 3). In more detail, moxidectin and Synriam achieved CRs of 14.8% (95% CI 0.04–0.3) and 11.1% (95% CI 0.02–0.3), and ERRs of 8.7% (95% CI -0.4–0.6) and 0% (95% CI -0.8–0.6), respectively. The two treatment arms containing praziquantel (Synriam plus praziquantel and praziquantel) yielded CRs of 60% (95% CI 0.4–0.8) and 38.5% (95% CI 0.2–0.6) and ERRs of 96% (95% CI 0.8–1.0) and 93.5% (95% CI 0.8–1.0), respectively.

**Table 3:** Effect of moxidectin, Synriam, Synriam plus praziquantel and praziquantel against *S*. *haematobium* and malaria co-infections. The study was carried out in the village of Moronou in central Côte d’Ivoire, between May and June 2015.

	Moxidectin (N = 27)	Synriam plus Praziquantel (N = 30)	Synriam (N = 27)	Praziquantel (N = 26)
***S*. *haematobium* infection**				
Children cured (%) (CI)	4 (14.8) (0.04–0.3)	18 (60) (0.4–0.8)	3 (11.1) (0.02–0.3)	10 (38.5) (0.2–0.6)
Children cured with high infection intensity (%)	0/8 (0)	3/9 (33.3)	0/8 (0)	2/7 (28.6)
Children cured with low infection intensity (%)	4/19 (21.1)	15/21 (71.4)	3/19 (15.8)	8/19 (42.1)
EPG before treatment AM	56.3	54	52.7	47
EPG after treatment AM	117.6	2.8	83.8	2.07
EPG before treatment GM	17.2	16	16.1	15.2
EPG after treatment GM	15.7	0.6	17.7	0.98
Egg reduction rate (%) (95% CI)	8.7 (-0.4–0.6)	96 (0.8–1.0)	0 (-0.8–0.6)	93.5 (0.8–1.0)
Microhematuria positive before treatment N (%)	16 (61.5)	13 (44.8)	14 (53.8)	12 (46.2)
Microhematuria negative after treatment N (%)	8 (29.6)	19 (63.3)	9 (33.3)	15 (57.7)
***Plasmodium falciparum* infection**				
Number RDT positive before treatment (%)	18 (66.7)	20 (66.7)	21 (77.8)	21 (80.7)
Number children negative based on RDT (%)	10/18 (55.5)	20/20 (100.0)	20/21 (95.0)	14/21 (66.6)
No. malaria direct smear positive before treatment (%)	18 (66.7)	20 (66.7)	17 (63.0)	19 (73.1)
No. children negative based on malaria smear (%)	10/18 (55.5)	20/20 (100.0)	17/17 (100.0)	12/19 (63.2)

CI, confidence interval; AM, arithmetic mean; GM, geometric mean, EPG, eggs per gram, RDT, rapid diagnostic test

Results of the *S*. *mansoni* cohort are reported in Table 4. ERRs were as follows: moxidectin 70.9% (95% CI 0.4–0.9), Synriam 64.9% (95% CI 0.4–0.8), Synriam plus praziquantel 77.6% (95% CI 0.5–1.1), and praziquantel 87.5% (95% CI 0.8–1.0). The CRs based on Kato-Katz were 12.9% (95% CI 0.03–0.3) for moxidectin, 6.7% (95% CI 0.01–0.2) for Synriam, 27.0% (95% CI 0.1–0.5) for Synriam plus praziquantel, and 27.6% (95% CI 0.1–0.5) for praziquantel. Praziquantel showed a moderate CR of 50.0% in adolescents harboring a low intensity infection. CRs according to CCA (trace results considered as positive) were 17.9%, 30.4%, 20%, 14.3% for moxidectin, Synriam plus praziquantel, Synriam, and praziquantel, respectively.

**Table 4:** Effect of moxidectin, Synriam, Synriam plus praziquantel and praziquantel against *S*. *mansoni* and malaria co-infections. The study was carried out in the villages of Bigouin and Biakalé in western Côte d’Ivoire, between May and June 2015.

	Moxidectin (N = 31)	Synriam plus Praziquantel (N = 26)	Synriam (N = 30)	Praziquantel (N = 29)
***S*. *mansoni* infection**				
Children cured (%) (95% CI)	4 (12.9) (0.03–0.3)	7 (27.0) (0.1–0.5)	2 (6.7) (0.01–0.2)	8 (27.6) (0.1–0.5)
Children cured with high infection intensity (%)	0/3 (0)	2/6 (33.3)	0/4 (0)	1/5 (20.0)
Children cured with moderate infection intensity (%)	2/13 (15.4)	2/9 (22.2)	0/13 (0)	1/12 (8.3)
Children cured with low infection intensity (%)	2/15 (13.3)	3/11 (27.3)	2/13 (15.4)	6/12 (50.0)
EPG before treatment AM	216.9	272.7	201.6	221.6
EPG after treatment AM	159.3	176.3	101	121.6
EPG before treatment GM	106.7	108.4	107	143.6
EPG after treatment GM	33.5	24.3	37.4	17.9
Egg reduction rate (%) (95% CI)	70.9 (0.4–0.9)	77.6 (0.5–1.1)	64.9 (0.4–0.8)	87.5 (0.8–1)
CCA positive (tr+) before treatment N (%)	28 (93.3)	23 (88.5)	25 (89.3)	28 (96.5)
CCA positive (tr-) before treatment N (%)	26 (86.6)	20 (76.9)	23 (82.0)	28 (96.5)
CCA negative (tr+) after treatment N (%)	5 (17.9)	7 (30.4)	5 (20.0)	4 (14.3)
CCA negative (tr-) after treatment N (%)	7 (27.0)	8 (40.0)	5 (21.7)	7 (25.0)
***Plasmodium falciparum* infection**				
No. RDT positive before treatment (%)	22 (71.0)	20 (77.0)	24 (80.0)	23 (79.0)
No. children negative based on RDT (%)	1 (4.5)	16 (80.0)	23 (95.8)	5 (21.7)

CI, confidence interval; AM, arithmetic mean; GM, geometric mean, EPG, eggs per gram; tr+, trace considered as positive; tr-, trace considered as negative; RDT, rapid diagnostic test

### Efficacy against co-infections

In the *S*. *haematobium* cohort all subjects in both Synriam treatment groups were cured from malaria infection according to direct smear technique, 5% (1/21) remained positive according to RDT. At follow up 55.5% of subjects in the moxidectin group were *Plasmodium* spp. negative based on both techniques. In the praziquantel treatment group 66.6% and 63.2% of participants were negative according to RDT and direct smear, respectively.

In the *S*. *mansoni* cohort overall 50% of subjects were *Plasmodium* spp. negative at follow up. CR in the groups treated with Synriam (praziquantel plus Synriam, Synriam respectively) were 80% and 95.8%.

In both cohorts none of the treatments showed any efficacy against STH infections (data not shown).

### Tolerability

At clinical examination all 128 subjects in the *S*. *haematobium* cohort reported symptoms. The number of adverse events stratified by treatment arm and evaluation time point are summarized in Table 5. Overall, recorded clinical symptoms decreased from 100% prior to treatment to 48% (3 hours post-treatment) and 21% (72 hours post-treatment). Three days after the last treatment administered, none of the participants reported any adverse events. The highest number of mild adverse events 3 hours post-treatment were recorded in the praziquantel treatment group (58%) and the lowest number in the Synriam plus praziquantel treatment group (41%). The most commonly observed adverse events were stomach ache (30%) and headache (19%) (S1 Table).

**Table 5:** Number of adolescents with clinical symptoms observed prior to treatment and adverse events in the *S*. *haematobium* study among the four different treatment arms, assessed at different time points. The study was carried out in the villages of Moronou, Bigouin and Biakalé in central Côte d’Ivoire, between May and June 2015.

Evaluation time points	Moxidectin (N = 31)	Synriam plus praziquantel (N = 32)	Synriam (N = 33)	Praziquantel (N = 32)	Overall (n = 128)
Clinical symptoms before treatment	31 (100.0)	32 (100.0)	33 (100.0)	32 (100.0)	128 (100.0)
3 hours after first treatment	14 (45.0)	13 (41.0)	17 (52.0)	18 (58.0)	62 (48.4)
24 hours after first treatment	9 (29.0)	9 (28.0)	4 (12.0)	6 (19.0)	28 (21.8)
72 hours after first treatment	7 (23.0)	9 (28.0)	6 (18.0)	5 (16.0)	27 (21.1)
3 hours after second treatment	NA	3 (9.0)	1 (3.0)	NA	4/65 (6.2)
24 hours after second treatment	NA	1 (3.0)	0 (0)	NA	1/65 (1.5)
72 hours after second treatment	NA	0 (0)	0 (0)	NA	0/65 (0)
3 hours after third treatment	NA	1 (3.0)	0 (0)	NA	1/65 (1.5)
24 hours after third treatment	NA	1 (3.0)	0 (0)	NA	1/65 (1.5)
72 hours after third treatment	NA	0 (0)	0 (0)	NA	0/65 (0)

In the *S*. *mansoni* cohort 95 subjects reported symptoms (74%) at clinical examination (Table 6). Overall, 51% of participants had mild symptoms at the first evaluation time point, ranging from 44% in the moxidectin group to 57% in the praziquantel treatment group. 15% of participants had symptoms 72 hours post-treatment and 3 days after the last treatment dose none of the participants reported symptoms.

**Table 6:** Number of adolescents with clinical symptoms observed prior to treatment and adverse events in the *S*. *mansoni* study among the four different treatment arms, assessed at different time points. The study was carried out in the villages of Moronou, Bigouin and Biakalé in central Côte d’Ivoire, between May and June 2015.

Evaluation time points	Moxidectin (N = 34)	Synriam plus praziquantel (N = 30)	Synriam (N = 32)	Praziquantel (N = 32)	Overall (N = 128)
Clinical symptoms before treatment	26 (76.0)	22 (73.0)	26 (81.0)	21 (65.0)	95 (74.2)
3 hours after first treatment	15 (44.0)	17 (57.0)	16 (50.0)	17 (53.0)	65 (50.7)
24 hours after first treatment	10 (29.0)	9 (30.0)	2 (6.0)	6 (19.0)	27 (21.1)
72 hours after first treatment	7 (21.0)	6 (20.0)	3 (9.0)	3 (9.0)	19 (14.8)
3 hours after second treatment	NA	1 (3.0)	0 (0)	NA	1/62 (1.6)
24 hours after second treatment	NA	1 (3.0)	0 (0)	NA	1/62 (1.6)
72 hours after second treatment	NA	1 (3.0)	0 (0)	NA	1/62 (1.6)
3 hours after third treatment	NA	1 (3.0)	0 (0)	NA	1/62 (1.6)
24 hours after third treatment	NA	1 (3.0)	1 (3.0)	NA	2/62 (3.2)
72 hours after third treatment	NA	0 (0)	0 (0)	NA	0/62 (0)

All events reported were mild, the most common symptoms were stomach ache (29%) and headache (10%). Only one subject had a moderate adverse event, namely eye swelling, which was resolved following antihistamine treatment. S1 Table and S2 Table summarize clinical symptoms stratified by treatment arm and assessment time for the *S*. *haematobium* and *S*. *mansoni* studies.

## Discussion

To our knowledge we have for the first time assessed the efficacy of Synriam and moxidectin against *S*. *mansoni* and *S*. *haematobium* infections. It is crucial to develop alternatives to praziquantel for the treatment of schistosomiasis, but lately no new drug candidates entered the drug discovery and development pipeline for this NTD [6]. Repurposing of existing drugs with different treatment indications as in the present exploratory trial, might be the way forward in order to find alternatives to praziquantel in a fast and cost-effective manner [27].

Based on promising findings in preclinical or clinical studies, two drugs were selected for our study, Synriam a new generation antimalarial drug and moxidectin, which will soon be marketed for the treatment of onchocerciasis [19, 28]. Disappointingly, we observed low efficacy of moxidectin and Synriam in terms of ERR and CR against *S*. *haematobium* though infection intensities in study participants were low. The highest CR and ERR (60% and 96%) were observed for Synriam plus praziquantel treated participants. Though the drugs were not administered simultaneously since possible drug interactions have not been studied to date, both treatments might have positively influenced each other. It could be hypothesized that the damage caused by praziquantel on *S*. *haematobium* was exacerbated by treatment with Synriam, which resulted in a slightly better efficacy when the two drugs were administered together. However, our study was not powered to detect statistical differences among treatment arms, hence studies with larger samples sizes would be necessary to confirm this finding.

Interestingly, against *S*. *mansoni* both moxidectin and Synriam alone performed better than against *S*. *haematobium* with higher ERRs observed but not CRs. The better efficacy of moxidectin and Synriam against *S*. *mansoni* in terms of ERRs cannot be explained at the moment. Fluctuations in egg counts might also play a role. However, the ERR observed for moxidectin in the present study is in line with previous results on *S*. *mansoni* [18]. In contrast with our findings in *S*. *haematobium* treated adolescents, in the *S*. *mansoni* study no increased efficacy was observed with Synriam plus praziquantel over praziquantel alone.

The findings on praziquantel observed in the two trials warrant further discussion. First, observed ERRs of praziquantel were similar in both cohorts (87.5–93.5%) and in accordance with previous findings [5, 29]. However, strikingly, praziquantel showed low CRs in participants infected with *S*. *haematobium* (38.4%) and *S*. *mansoni* (27.6%). These CRs are notably lower (except for one of our own studies in a nearby setting [5]) than in previous reports, which have documented CRs (for a single dose of praziquantel (40 mg/kg)) ranging from 51 to 100% against *S*. *mansoni* and *S*. *haematobium*. Our findings underline the great variability of CRs linked to praziquantel treatment [30]. As discussed elsewhere [31,32] the low CRs of praziquantel in the *S*. *mansoni* cohort might be due to the fact that half of participants suffered from moderate/heavy infection intensity. Praziquantel revealed a higher CR of 50% in adolescents with low intensity *S*. *mansoni* infections. Similarly, a higher CR (42%) was observed in participants with a low *S*. *haematobium* infection intensity.

Many clinical trials have been conducted using artemether and artesunate alone and in combination with praziquantel [33]. The moderate efficacies against *S*. *mansoni* and *S*. *haematobium* observed with the antimalarial Synriam in our trial are in line with previous studies using peroxidic drugs as monotherapy against chronic schistosome infections [34, 35]. For example, a recent meta-analysis determined that artesunate has a low CR against chronic *S*. *haematobium* infection (25%) [34, 36]. This finding is not surprising since artemisinin derivatives mainly act on juvenile schistomes [8]. We had therefore initially planned a second follow up at 50 (*S*. *mansoni*) and 80 days (*S*. *haematobium*) after treatment, to assess the efficacy of Synriam against prepatent infections. However, this additional survey was not conducted, due to the low efficacy observed at the first follow up, which made it impossible to assess activity against juvenile schistosome infections. Follow up studies could be planned to assess the prophylactic effect of Synriam and Synriam plus praziquantel against infections with *S*. *mansoni* and *S*. *haematobium*.

In the *S*. *haematobium* cohort we used both filtration and assessment of microhaematuria. Our data confirm [37] that the evaluation of microhematuria for the diagnosis of *S*. *haematobium* has a lower sensitivity compared to the filtration method. However, urine filtration was performed on three samples while microhaematuria was investigated on only one. Furthermore, the majority of participants in our study had low *S*. *haematobium* intensity infections.

In both settings we examined *Plasmodium* infections: based on RDT in both cohorts infected adolescents treated with Synriam were all cured with the exception of four patients (CRs 80–100%). CRs with the thin and thick smears were slightly lower. To date few studies have been conducted with Synriam against *Plasmodium* infections: two on the African and Asian continents reported CRs of 90–99% 28 days after treatment, a second follow up was performed 42 days after the last dose of drug and 100% of participants had no gametocytes [38]. The rate of reinfection was lower than 0.5% at 28 days [38]. Similar results were found in another study comparing artemether plus lumefantrine and Synriam, with all patients being cured 28 days post-treatment [39]. The moderate CRs observed in the praziquantel and moxidectin treated groups in the *S*. *haematobium* setting cannot fully be elucidated. Spontaneous clearance might have occurred, however we also cannot rule out self-treatments.

Interestingly, in both trials a higher number of clinical symptoms was observed before treatment compared to post-treatment. For example, before treatment 41% (*S*. *haematobium* cohort) and 54% (*S*. *mansoni* cohort) participants reported headache, whereas 3 hours after treatment only 19% and 10% reported this symptom in the two cohorts, respectively. A similar finding was observed for stomach ache, which rapidly improved after treatment. These results are contradictory to most studies, which observe a worsening of gastrointestinal symptoms following treatment with praziquantel most likely due to dying worms [29]. We cannot fully explain our findings, however it might be worth highlighting that the number of participants complaining about symptoms prior to treatment was very high, higher than in previous studies, which makes it difficult to present a clear picture on the occurrence of adverse events. In addition, it is not possible to distinguish between an actual improvement in their conditions (which however seems to have occurred too fast) or a perceived improvement, driven by participants' expectations or other factors.

In conclusion, the two drugs tested (moxidectin and Synriam) showed low efficacy against *S*. *haematobium* infections. We therefore do not expect a significant ancillary benefit when these two drugs are used for the treatment of onchocerciasis and malaria in settings co-endemic for *S*. *haematobium*. We observed a better performance of both drugs in terms of ERR against *S*. *mansoni* infection. Studies with larger sample size are needed to confirm our finding and to rule out that these results have occurred by chance.

## Supporting Information

S1 Checklist Consort checklist. (PDF)

S1 Protocol Trial protocol. (PDF)

S1 Table Number of adolescents with clinical symptoms prior to treatment and adverse events among the four different treatment arms assessed at different time points in the *S*. *haematobium* cohort. (DOCX)

S2 Table Number of adolescents with clinical symptoms prior to treatment and with adverse events among the four different treatment arms assessed at different time points in the *S*. *mansoni* cohort. (DOCX)
