# Supplementary material for: Efficacy and Safety of Moxidectin, Synriam, Synriam-Praziquantel versus Praziquantel against Schistosoma haematobium and S. mansoni Infections: A Randomized, Exploratory Phase 2 Trial
Source: PLoS Negl Trop Dis. 2016 Sep 16;10(9):e0005008. doi: 10.1371/journal.pntd.0005008 (PMC5026339; doi:10.1371/journal.pntd.0005008)

Study Protocol

Assessment of the safety and efficacy of oral Moxidectin, Synriam®, Synriam®-Praziquantel combination versus Praziquantel in schoolchildren infected with *Schistosoma haematobium* and *Schistosoma mansoni*

**Protocol Number**	1		
**Version Number**	1.01	**Document Date**	21.10.2014
**Sponsor Contact**	Prof. Dr. Jennifer Keiser, Swiss Tropical and Public Health Institute, Tel.: +41 61 284-8218<br>Fax: +41 61 284-8105<br>E-mail: jennifer.keiser@unibas.ch		
**Principle Investigator**	Prof. Dr. Jennifer Keiser, Swiss Tropical and Public Health Institute, Tel.: +41 61 284-8218<br>Fax: +41 61 284-8105<br>E-mail: jennifer.keiser@unibas.ch		
**Funding Agency**	Geigy Foundation, ERC		

1. General Information

I. List of investigators and other persons involved

Title	Names	Institution	Position	Function in trial
Prof. Dr.	Jennifer Keiser	Swiss Tropical and Public Health Institute	Unit head	Principal Investigator
Dr	Beatrice Barda	Swiss Tropical and Public Health Institute	PhD student	Co-PI
Dr.	Jean Coulibaly	CSRS/University Félix Houphouët Boigny	Project leader	Co-PI
Dr.	Jan Hattendorf	Swiss Tropical and Public Health Institute	Project leader	Statistician
Prof	N'Goran K. Eliézer	CSRS/University Félix Houphouët Boigny		Advisor

II. Signatures

Statistician

Signature		Date of Signature<br>21.10.2014
Name	Jan Hattendorf	
Title	Dr.	
Institution	Swiss Tropical and Public Health Institute	
Address	Department of Medical Parasitology and Infection Biology<br>Swiss Tropical and Public Health Institute, Socinstr. 57<br>CH- 4002 Basel, Switzerland	
Phone	+41 61 284-8193	

Principle investigator(s)

I have read this protocol and agree that it contains all necessary details for carrying out this trial. I will conduct the trial as outlined herein and will complete the trial within the designated time. I will provide copies of the protocol and all pertinent information to all individuals responsible to me who assist in the conduct of this trial. I will discuss this material with them to ensure they are fully informed regarding the drug and the conduct of the trial.

I will use only the informed consent forms approved by the Sponsor or its representatives and will fulfil all responsibilities for submitting pertinent information to the Independent Ethics Committees responsible for this trial.

I agree that the Sponsor or its representatives shall have access to any source documents from which Case Report Form information may have been generated.

Principle investigator

Signature		Date of Signature
Name	Jennifer Keiser	
Title	Prof.	
Institution	Swiss Tropical and Public Health Institute	
Address	Department of Medical Parasitology and Infection Biology<br>Swiss Tropical and Public Health Institute, Socinstr. 57<br>CH- 4002 Basel, Switzerland	
Phone	+41 61 284-8218	

Co-Principle investigator

Signature		Date of Signature
Name	Beatrice Barda	
Title	MD, PhD student	
Institution	Swiss Tropical and Public Health Institute	
Address	Department of Medical Parasitology and Infection Biology<br>Swiss Tropical and Public Health Institute, Socinstr. 57<br>CH- 4002 Basel, Switzerland	
Phone	+41 61 284 82 86	

Co-Principle investigator

Signature		Date of Signature
Name	Jean Coulibaly	
Title	Dr	
Institution	CSRS, University Félix Houphouët Boigny	
Address	22 BP 770, Abidjan 22, Côte d'Ivoire	
Phone	+225 0500 8223	

III. Table of contents

1. General Information 2
2. Background information 11
3. Trial objective and purpose 14
4. Trial design 15
4.1 Primary and secondary endpoint 15
4.2. Type of trial 15
4.3. Trial design 15
4.3.1 Baseline survey 15
4.3.2 Assessment of efficacy after treatment 15
4.4. Measure to minimize bias 16
4.5. Study duration and duration of subject participation 16
4.6. Schedule of visits 17
5. Selection of the trial subjects 18
5.1 Recruitment 18
5.2 Inclusion criteria 18
5.3. Exclusion criteria 18
5.4. Criteria for discontinuation of trial 19
5.5. Treatment of subjects 19
5.6 Concomitant therapy 20
6. Assessment of safety 21
6.1. Safety parameters and collection and reporting of adverse events 21
6.1.1. Types of adverse events 21
6.1.2. Serious adverse events 22
6.1.3. Serious adverse event reporting 23
7. Statistics 24
7.1. Definition of primary endpoint 24
7.2. Justification of number of trial subjects 24
7.3. Description of statistical methods 24
7.4. Description of data management 25
8. Duties of the investigator 25
8.1. Investigator's confirmation 25
8.2. Damage coverage 26
8.3. Project management 26
9. Ethical considerations 26
9.1. Independent Ethics Committee (IEC) 26
9.2. Evaluation of the risk-benefit ratio 27
9.3. Subject information and consent 27
9.4. Subject Confidentiality 27
9.5. Subjects requiring particular protection 27
9.6. Other aspects .... **Error! Bookmark not defined.**
10. Quality control and quality assurance 28
10.1. Monitoring and auditing 28
10.2. Access to data, handling of data and samples (data protection), archiving (place, duration) and destruction 28
10.3. Data entered directly in the Case Report Form (CRF) – definition of source data 29
10.4. Data and safety monitoring board (WHO) / data monitoring committee (EU/FDA) 29
11. Dissemination of results and publication 29
12. References 30

IV. Synopsis

**Study Title**	Assessment of the safety and efficacy of oral Moxidectin, Synriam® and Synriam®-Praziquantel combination versus praziquantel in schoolchildren infected with *Schistosoma haematobium* and *Schistosoma mansoni*
**Study Phase**	2a
**Indication**	*S. mansoni* (study 1) and *S. haematobium* (study 2) infection (eggs in stool/urine) and malaria infection
**Investigational Product and Reference Treatment**	Moxidectin, Synriam® and Synriam®-Praziquantel
**Protocol Number, Date and Version**	1, 25.11.2014, V1.01
**Trial registration**	not yet available
**Study Rationale**	Assess for the first time the safety and efficacy of oral moxidectin, Synriam® and Synriam®-praziquantel compared to praziquantel on *S. mansoni* and *S. haematobium* infection in schoolchildren
**Study Objectives**	The **primary objective** of the trial is to test the efficacy (cure rate (CR) and egg reduction rate (ERR)) of moxidectin and Synriam® (Arterolane (OZ277) and piperaquine) and a Synriam®-praziquantel combination compared to praziquantel against schistosome infections.<br><br>The **secondary objectives** of the trial are:<br>a.) Evaluate the safety of the treatment regimen in school-aged children<br>b.) To determine the CR and ERR of Moxidectin, Synriam® and Synriam®/Praziquantel combination against possible co-infections (*Ascaris lumbricoides*, *Trichuris trichiura,* hookworm, *Strongyloides stercoralis*)<br>c.) To determine the efficacy of Synriam® against malaria infection
**Study design**	Randomized, phase 2a, drug efficacy trial
**Study product / intervention**	Moxidectin, Synriam®, Synriam®/Praziquantel

**Comparator(s)**	Praziquantel
**Key inclusion / Exclusion criteria**	Inclusion: Children infected with *S. mansoni* (study 1) and *S. haematobium* (study 2), absence of major systemic illnesses, written informed consent signed by parents and/or legal guardian; and oral assent by children.<br>Exclusion: Any abnormal medical conditions or chronic disease, negative diagnostic result for schistosome infections, no written informed consent, recent anthelminthic or antimalarial treatment (past 3 months)
**Primary Endpoints**	Safety, CR and ERR of moxidectin and Synriam® and Synriam®/praziquantel against *S. mansoni* and *S. haematobium*
**Secondary Endpoints**	CR and ERR of Moxidectin and Synriam® against possible co-infections (*A. lumbricoides*, *T. trichiura*, hookworms, *S. stercoralis)*<br>Efficacy of Synriam® against malaria infection
**Exploratory Endpoints**	None
**Interim Analyses**	None
**Study Duration**	80 days
**Schedule**	05/2015 of first-participant in (planned)<br>08/2015 of last-participant out (planned)
**Measurements & procedures**	Two stool samples (study 1), three urine samples (study 2) and one blood finger prick sample will be collected if possible on two consecutive days or otherwise within a maximum of 5 days. The medical history of the participating schoolchildren will be assessed with a standardized and previously used questionnaire, in addition to a clinical examination carried out by the study physician on the treatment day. School-aged children will also be interviewed before treatment, 2 and 24 hours after treatment about the occurrence of adverse events. The efficacy of the treatment will be determined 21 and 50/80 days post-treatment by collecting other two stool samples or three urines and one finger prick. All stool samples will be examined with duplicate Kato-Katz thick smears and Baermann method for the detection of *S. stercoralis;* all urine samples will be analyzed with filtration method for *S. haematobium* eggs and reagent strips for appraisal of microhaematuria and CCA diagnostic test. All finger pricks will be analyzed with thick and thin smear for appraisal of malaria parasitaemia and a rapid malaria diagnostic test.

**Statistical Analyses**	An available case analysis will be performed, including all children with primary end point data. Supplementary, a per-protocol analysis and an intention-to-treat analysis will be conducted. CRs will be calculated as the percentage of egg-positive children at baseline who become egg-negative after treatment. Geometric and arithmetic mean egg counts will be calculated for the different treatment arms before and after treatment to assess the corresponding ERRs Bootstrap resampling method with 2,000 replicates will be used to calculate 95% confidence intervals (CIs) for ERRs. Differences in ERRs will be determined under the assumption that non-overlapping CIs indicate statistical significance.
**GCP statement**	This study will be conducted in compliance with the protocol, the current version of the Declaration of Helsinki, ICH-GCP E6 as well as all national legal and regulatory requirements.
**Key explanation for the inclusion of children**	This study will be carried out in school-aged children, since infections with schistosomes occur most often in children, hence this age group is at highest risk of infections.
**Recruitment procedure**	The study will be carried out in school-aged children (age: 6-14 years) of two primary schools (CNRA and Moapé) in Azaguié and Adzopé, Côte d'Ivoire in two areas that are highly endemic for *S. mansoni* and *S. haematobium*, respectively.
**Coverage of damages**	Winterthur Police Nr. 4746321, GNA Assurance
**Storage of data and samples for future research aims**	After the study has been completed all samples will be destroyed and case report forms will be kept for a minimum of 10 years (chapter 10)

2. Background information

Known since ancient times, schistosomiasis (bilharzia) belongs to one of the most neglected tropical diseases, although it is affected by great morbidity with significant economic and public health consequences [1]. Schistosomiasis is caused by one of six species of schistosomes, with *Schistosoma mansoni, S. japonicum*, and *S. haematobium* being the most prevalent species [2]. It is currently estimated that approximately 230 million people are infected [2] and approximately 11,000 people die from the disease each year [1]. Millions of people suffer from severe disease manifestations including obstructive uropathy and bladder calcification (*S. haematobium*) and periportal hepatic fibrosis (*S. mansoni* and *S. japonicum*). The disease gives rise to a persistent chronic disorder in endemic areas, resulting in common disabling complications such as anaemia, growth stunting, cognitive impairment, and decreased aerobic capacity [2].

Control of schistosomiasis is based on preventive chemotherapy interventions targeting the entire at-risk population. At the World Health Assembly (WHA) in 2001, resolution no. 54.19 was put forward which urged endemic countries to start seriously tackling worms, specifically schistosomiasis and soil transmitted helminthiases (STH), with a global target to treat at least 75% of all school aged children who are at risk of morbidity from schistosomiasis and STH by the year 2010 [3]. Control of all forms of schistosomiasis is currently dependent on a single drug, praziquantel. Praziquantel was developed in the early 1970s for veterinary practice, as it is true of all available anthelmintics for humans [4]. Praziquantel is active against adult schistosomes, but has little activity against the juvenile schistosomula, the young developmental stages of the parasite [4]. The high drug pressure from the widespread administration of praziquantel could lead to problematic drug resistance [5]. Should serious praziquantel resistance arise, there are no viable alternatives to this [6]. Even so, drug discovery for schistosomiasis has languished, although several antischistosomal lead compounds have been identified in recent years. Nonetheless, since no drug is currently undergoing clinical testing for schistosomiasis [7], a backup drug for praziquantel will be not available for the next decade.

The discovery of artemisinin, the active constituent of the herb Artemisia annua, was one of the most important breakthroughs in malaria chemotherapy [8]. Artemisinin is a sesquiterpene lactone that contains a peroxide bond in a unique 1,2,4-trioxane heterocycle. The semisynthetic artemisinins also have antischistosomal properties as widely evaluated in laboratory studies and clinical trials [9]. In retrospect, it might not be so surprising that the semisynthetic artemisinins (and other peroxidic compounds) possess both antimalarial and

antischistosomal activities, as both plasmodia and schistosomes digest hemoglobin. The success of the semi-synthetic artemisinins along with their pharmacologic draw-backs motivated the creation of fully synthetic derivatives in antimalarial drug discovery, the most investigated of these being the synthetic ozonides. At Swiss TPH we discovered that several ozonides also have significant antischistosomal potential. The first of these was ozonide OZ78, which, like the semisynthetic artemisinins, has high activity against the juvenile stage of *S. mansoni* [10]. In contrast to the lack of activity of OZ78 against adult stage *S. mansoni* in the mouse model, this ozonide had high efficacy against adult worms in *S. mansoni*- and *S. japonicum*-infected hamsters [11]. The mechanistic basis for the higher efficacy of OZ78 (and other peroxides) in schistosome-infected hamsters vs. mice is unclear. The first ozonide antimalarial, Synriam® (arterolane (OZ277) and piperaquine) (Ranbaxy®) has been launched a few months ago and there is a need to study its antischistosomal activities in an exploratory clinical trial [12]. The pediatric dosage is under investigation and has now entered phase III clinical trial [13], the tablet formulation used is arterolane 37.5 mg/ piperaquine 187.5 mg and the dose is adjusted according to age between 6 months and 12 years old.

Moxidectin is a macrocyclic lactone derived from the actinomycete *Streptomyces cyanogriseus* spp. *noncyanogenus* used in veterinary practice since 1985 [14]. Its main use was against lymphatic filariasis, but was demonstrated to be effective against intestinal parasites as well [15]. Studies conducted in vitro have demonstrated that moxidectin was more efficacious than ivermectin both in vivo and in vitro [16]. Considering these promising results, it has recently been adapted by human medicine as well for the treatment of onchocerciasis with good results in terms of efficacy against microfilariae and safety [17, 18]. The rationale behind the research for an alternative to ivermectin in the treatment of lymphatic filariasis was to find a drug for mass treatment for onchocerciasis control with a good efficacy in interrupting the transmission of *O. volvulus.* The filaricidal mechanism of moxidectin is still under investigation, but studies on its kinetic have demonstrated that it influences the glutamate-gated chloride channels and the gamma-aminobutyric acid receptor complex and consequently having a paralytic effect and death of the parasite [19, 20]. The bioavailability of the drug in oral formulation demonstrated in rats was moderate and the $t_{1/2\ elim}$ ranged between 22.9 and 44.6 hours; further studies conducted on different animals showed that the maximal concentration of oral moxidectin is reached 2 to 9 hours after administration [17]. Studies conducted on moxidectin pharmacokinetic in humans have showed that either the liquid or tablet formulation are quickly absorbed [21], extensively distributed and has a long half-life. Korth-Bradley at *al*. have seen an increased bioavailability of the drug by the simultaneous consumption of food [22].

These promising results have lead Attah et *al*. to conduct a preliminary study of efficacy of moxidectin against intestinal parasites; their results show a good efficacy of the drug especially against *T. trichiura,* hookworms, *A. lumbricoides* and *S. mansoni* compared to ivermectin [23]. Given these interesting data further studies are necessary to assess the efficacy and safety of moxidectin in the treatment of schistosomes and soil-transmitted helminth infections.

3. Trial objective and purpose

To compare the efficacy and safety of the following new drugs and drug combinations in school-aged children infected with *S. mansoni* (study 1) and *S. haematobium* (study 2):

i) Moxidectin liquid formulation 8 ml (8 mg), ii) Synriam® (112.5 mg arterolane+ 562.5 mg piperaquine (age 6-12 years)/ 150 mg arterolane+ 750 piperaquine (age 12-14 years) for three consecutive days, iii) Synriam® (112.5 mg arterolane+ 562.5 mg piperaquine (age 6-12 years)/ 150 mg arterolane+ 750 piperaquine (age 12-14 years)) for three consecutive days+ praziquantel 40 mg/kg single dose; iv) Praziquantel 40 mg/kg single dose

The **primary objective** of the trial is to determine the efficacy of moxidectin and Synriam® (Arterolane (OZ277) and piperaquine) and a Synriam®-praziquantel combination compared to praziquantel against *S. mansoni* and *S. haematobium* infections

.

The **secondary objectives** of the trial are:

a.) Evaluate the safety of the treatment regimen in school-aged children

b.) To determine the CR and ERR of moxidectin, Synriam® and Synriam®/praziquantel combination against possible co-infections (*Ascaris lumbricoides*, *Trichuris trichiura,* hookworm *Strongyloides stercoralis*)

c.) To determine the efficacy of Synriam® against malaria infection

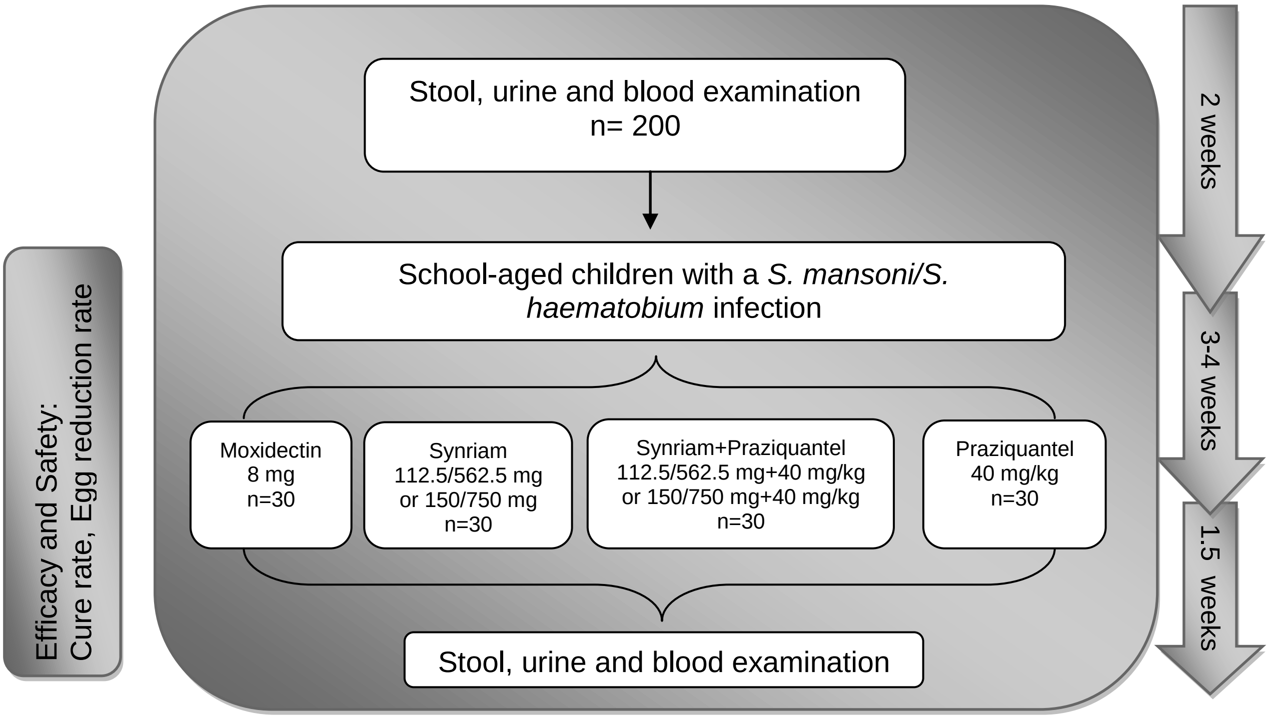

Figure 1: Administration of different doses of moxidectin and different combinations of Synriam® study.

4. Trial design

4.1 Primary and secondary endpoint

CR (i.e. conversion from being egg positive pre-treatment to egg negative post-treatment) and ERR (primary end points) against *S. mansoni* and *S. haematobium* as well as clinical parameters and CR and ERR against concomitant helminth infections (secondary endpoint). CR as well as clinical parameters against *Plasmodium spp* infection

4.2. Type of trial

Randomized, controlled phase 2a efficacy trial.

4.3. Trial design

4.3.1 Baseline survey

The medical history of school–aged children participating in the study will be assessed with a standardized and previously used questionnaire, in addition to a clinical examination carried out by the study clinician. Two stool samples (*S. mansoni* study), three urine samples (*S. haematobium* study) and one blood finger prick sample will be collected from school-aged children until 120 cases of *S. mansoni* and *S. haematobium* infections have been identified (30 cases per treatment arm), regardless to concomitant infections with *A. lumbricoides, T. trichiura, S. stercoralis* and hookworm. The expected prevalence for schistosome positive school children was estimated to be 60% [24,25]. Hence, we anticipate enrolling approximately 200 children in each study site (to yield at least 120 *S. mansoni* and *S. haematobium*-positive children).

The standard urine filtration method (10 ml of urine) will be used for appraisal of *S. haematobium* eggs [26]. The Kato-Katz technique will be used for the quantitative assessment of *S. mansoni* infections [27]. Baermann technique will be used for the quantitative detection of *S. stercoralis* larvae [28]. Each child will be invited to provide 3 urine (*S. haematobium* study) and 2 stool samples (*S. mansoni* study) within a maximum of 5 days. In addition, we will use reagent strips for appraisal of microhaematuria and the CCA (will be obtained from Govert van Dam, Leiden) [29] diagnostic tests; urine and stools samples might be preserved for possible additional diagnostic tests. In addition, one stool sample will be collected from each child in the *S. haematobium* study and one urine sample from each child participating in the *S. mansoni* study. Infection intensity (expressed as the arithmetic mean egg count per gram of stool (epg)/per 10 ml of urine will be calculated for

each individual. Infections with soil-transmitted helminths, i.e. *A. lumbricoides*, hookworm and *T. trichiura*, will also be assessed and recorded for each parasite species separately.

A finger prick blood sample will be taken, thick and thin blood smears prepared on a microscope slide for subsequent appraisal of malaria parasitaemia together with dosage of haemoglobin. All the slides will be double-checked by a second laboratory technician and only considered negative if no parasites detected in 100x oil immersion field by the two independent microscopists. Additionally, a rapid malaria diagnostic test will be employed

4.3.2 Assessment of efficacy after treatment

At day 21 and day 50 (*S. mansoni*) or day 80 (*S. haematobium* study) after the last treatment dose has been administered we will sample again 3 urine and 2 stool specimen for analysis of *S. haematobium, S. mansoni* and soil-transmitted helminth infections, together with a finger prick for the diagnosis of malaria infection and haemoglobin dosage. Infection intensity will be calculated for each individual and cure and egg reduction rates calculated. In addition, malaria parasitaemia will be assessed at both follow up time points. At the end of the study all participating children still positive for *S. mansoni, S. haematobium*, *A. lumbricoides*, *S. stercoralis,* hookworm and *T. trichiura* will be treated with albendazole and/or praziquantel and/or ivermectin and following national guidelines for malaria treatment [30,31].

4.4. Measure to minimize bias

Study participants eligible for treatment will be randomly assigned to one of the four treatment arms using a computer-generated stratified block randomization code. The random allocation sequence with varying random blocks of seven or fourteen will be provided by a statistician. The codes will be held in a locked cabinet at the Swiss Tropical and Public Health Institute. A copy of this code will be kept in a sealed envelope by one of the co-investigators (will only be opened in emergency situations, determined by the principal investigator upon consultation with the co-investigators). Laboratory personnel will not know which child was allocated to which treatment arm and will therefore be blinded.

4.5. Duration of study and subject participation

Each trial will last three months. The screening for the baseline will start one week prior to the treatment. Follow up screening will take place 21-22 and 50 and 80 days post-treatment and last for one week. Schedules of visits are summarized in the appendix.

4.6. Schedule of visits

Table 1 schedule of visits

	**Visit 1 Screening starting at day n-8**	**Visit 2 Baseline Day 0-3**	**Visit 3 24 hrs post-treatment**	**Visit 4 3 week post-treatment**	**Visit 5 7 or 11 weeks post-treatment**
ICF signature	x				
Collection of stool, urine and blood samples	x			x	x
Administration of drugs		x			
Stool, urine and blood examination	x			x	x
Physical examination	x	x	(x)	(x)	
Vital signs (BP, HR, Temperature, Hb)	x		x	x	x
Signs and symptoms, adverse events and concomitant medication		x	x		

5. Selection of the trial subjects

5.1 Recruitment

The study will be carried out in school-aged children (age: 6-14 years) of two primary schools (CNRA and Moapé) in Azaguié and Adzopé, Côte d'Ivoire, areas that are highly endemic for *S. mansoni* and *S. haematobium*, respectively [24,25].

The parents/guardians of the children will be invited to participate in an information meeting to explain the purpose and procedures of the study, including potential benefits and risks. Parents will be encouraged to ask questions in an open discussion forum.

5.2 Inclusion criteria

1. Written informed consent signed by parents and/or legal guardian; and oral assent by children.
2. Able and willing to be examined by a study physician at the beginning of the study.
3. Able and willing to provide two stool samples, three urines and one finger prick at the beginning (baseline) and approximately three weeks after treatment (follow-up).
4. Positive for *S. mansoni* or *S. haematobium* eggs in the stool and/or in urine.
5. Absence of major systemic illnesses (e.g. cancer, diabetes, clinical malaria or hepato-splenic schistosomiasis) as assessed by a medical doctor, upon initial clinical assessment.
6. No known or reported history of chronical illness as cancer, diabetes, chronic heart, liver or renal disease.
7. No recent anthelminthic or antimalarial treatments (within past 4 weeks).
8. No known allergy to study medications.

5.3. Exclusion criteria

1. No written informed consent by parents and/or legal guardian.
2. Presence of any abnormal medical condition, judged by the study physician.
3. History of acute or severe chronic disease such as cancer, diabetes, chronic heart, liver or renal disease.
4. Recent use of anthelminthic or antimalarial drugs (within past 4 weeks).
5. Attending other clinical trials during the study.
6. Negative diagnostic result for *S. mansoni* or *S. haematobium* (absence of helminth eggs in stool/urine).

Participants who were diagnosed with a soil-transmitted helminth or malaria infection, but who were excluded from the study due to one or several of the above-mentioned exclusion criteria, including withdrawals will be offered standard anthelminthic/antimalarial treatment (Albendazole/Praziquantel/Ivermectin and malaria treatment according to national guidelines).

5.4. Criteria for discontinuation of trial

A subject can be discontinued from the study for the following reasons:

1. Withdraws from the study (this can happen anytime as participation is voluntary and there are no further obligations once a child withdraws).
2. At the discretion of the Principal Investigator, if the participant is not compliant to the requirements of the protocol.

Discontinued subjects will not be replaced. If, for any reason, a subject is discontinued from the study before the end of treatment evaluations, the safety procedures planned (adverse events monitoring) will be conducted.

5.5. Treatment of subjects

Moxidectin will be obtained by Virbac Pharma industry (Nice, France) and Synriam® will be obtained from Ranbaxy (Gurgaon, India). The formulation of Synriam® will be either the adult (150/750 mg) or the pediatric one (37.5/187.5 mg) and dose adjusted according to the age of the child (6-12 years 112.5/ 562.5 mg and 12-14 years 150/750 mg) [13]. The subjects will be randomized into four different groups (Table 2).

Table 2: Treatment arms

	**Day 1**	**Day 2**	**Day 3**
**Group 1**	Praziquantel 40 mg/kg		
**Group 2**	Synriam 112.5/ 562.5 mg or 150/750 mg	Synriam 112.5/562.5 mg or 150/750 mg	Synriam 112.5/562.5 mg or 150/750 mg
**Group 3**	Praziquantel 40 mg/kg (morning), Synriam 112.5/562.5 mg or 150/750 mg (afternoon)*	Synriam 112.5/562.5 mg or 150/750 mg	Synriam 112.5/562.5 mg or 150/750 mg
**Group 4**	Moxidectin x 8 mg		

*praziquantel has a very short half life and if the two treatments are spaced by half a day drug interactions are not expected

All treatments will be administered in the presence of the investigator(s), and ingestion confirmed. This will be recorded with the time and date of closing. Subjects will be asked not to take any drugs other than those prescribed by the study medical team. After ingestion of the medication, the subjects will be observed for 3 hours to ensure retention of the drug. Vomiting within 1-hour post-dosing will require re-dosing. The subjects will not allow more than one repeated dose. No re-administration will be needed for subjects vomiting after one hour.

The Principal Investigator is responsible for drug accountability at the study site. Maintaining drug accountability includes careful and systematic study drug storage, handling, dispensing and documentation of administration.

5.6 Concomitant therapy

All medications taken one month before and during the study period must be recorded with indication, dose regimen, date and time of administration.

Medication(s)/treatment(s) permitted during the trial

- Analgesics and antipyretics are allowed to be given to the subjects in case of fever, antiemetic to prevent nausea and vomiting and/or antibiotics to prevent or treat bacterial superinfection.

Medication(s)/treatment(s) NOT permitted during the trial

- No other active drugs against helminthes/malaria are permitted during the trial

6. Assessment of safety

6.1. Safety parameters and collection and reporting of adverse events

Little adverse events have been reported following moxidectin, Synriam® and praziquantel administration. The most common adverse events were abdominal cramps, fever, nausea, headache, and vertigo [30,32]; in some subjects haematologic adverse events have been registered [17] and Mazzotti reaction in countries endemic for *O. volvulus* [18].

The observation time for adverse events starts when the treatment is initiated. Patients will be kept for observation for at least 3 hours following treatment for any acute adverse events. If there is any abnormal finding, the local study physician will perform a full clinical examination and findings will be recorded. An emergency kit will be available on site to treat any medical conditions that warrant urgent medical intervention. Severely-ill subjects will be referred to the nearby health center or hospital for prompt medical care. Patients will also be interviewed 24 hours after treatment about the occurrence of adverse events.

Information on all adverse events (incidence, onset, cessation, duration, intensity, frequency, seriousness and causality) will be entered immediately in the source document, and also in the appropriate adverse event module of the case report form (CRF). For all adverse events, sufficient information will be pursued and/or obtained so as to permit i) an adequate determination of the outcome of the event (i.e. whether the event should be classified as a serious adverse event); and; ii) an assessment of the casual relationship between the adverse event and the study treatments. Intensity of adverse events will be judged by the study physician, following guidelines by the European Medicine Agency (Note for Guidance on Clinical safety Data Management).

6.1.1. Types of adverse events

The term "adverse event" could include any of the following events which develop or increase in severity during the course of the study:

a) Any signs or symptoms whether thought to be related or unrelated to the condition under study;
b) Any clinically significant laboratory abnormality;
c) Any abnormality detected during physical examination.

These data will be recorded on the appropriate CRFs, regardless of whether they are thought to be associated with the study or the drug under investigation. Associated with the use of

the drug means that there is a reasonable possibility that the event may have been caused by the drug.

Adverse signs or symptoms will be graded by the Investigator as mild, moderate, severe or intolerable according to the following definitions: *(CTC or WHO grading scales facilitate AE reporting)*

Grade	Definition
Mild (1):	Causing no limitation of usual activities.
Moderate (2):	Causing some limitation of usual activities.
Severe (3):	Causing inability to carry out usual activities.
Intolerable (4):	Intolerable or life threatening.

The observation time for adverse events starts when the treatment is initiated and continues until discharge from the facility at the End of Treatment. For the purpose of this trial, disease progression and relapse will be considered as treatment failure, not as an Adverse Event.

Expected adverse event: Risks or events reported in the literature or by WHO (http://apps.who.int/medicinedocs/en/d/Jh2922e/3.1.4.html#Jh2922e.3.1.4) in the Ivestigator's Brochure and listed in the consent form.
Unexpected adverse event: Any adverse event, the frequency, specificity or severity of which is unanticipated and not consistent with the available risk information described for these drugs.

6.1.2. Serious adverse events

A "serious" adverse event is defined as any event that suggests a significant hazard, contraindication, side effect, or precaution. A serious adverse event includes any event that:

1. is fatal;
2. is life threatening, meaning, the subject was, in the view of the Investigator, at immediate risk of death from the reaction as it occurred, i.e., it does not include a reaction that, had it occurred in a more serious form, might have caused death;
3. is a persistent or significant disability or incapacity, i.e., the event causes a substantial disruption of a person's ability to conduct normal life functions;
4. requires, or prolongs in-patient hospitalization;
5. is a congenital anomaly or birth defect;

6. is an important medical event, based upon appropriate medical judgment, that may jeopardize the patient or subject or may require medical or surgical intervention to prevent one of the other outcomes defining serious.

An unexpected event is any adverse event that is not identified in nature, severity, or frequency in the scientific literature.

A “severe” adverse event does not necessarily meet the criteria for a “serious” adverse event.

Serious adverse events that are still ongoing at the end of the study period will be followed up to determine the final outcome. Any serious adverse event that occurs after the study period and is considered to be possibly related (definitions will be used according to the WHO-UMC system; see: http://who-umc.org/Graphics/24734.pdf) to the study treatment or study participation will be recorded and reported immediately.

6.1.3. Serious adverse event reporting

Any study-related unanticipated problem posing risk of harm to subjects or others, and any type of serious adverse event, will be reported to the study sponsor by telephone within 24 hours of the event. To report such events, a serious adverse event (SAE) form (CIOMS Form 1) must be completed by the investigator and faxed to the Swiss TPH within 24 hours. The co-investigator will keep a copy of this SAE form on file at the study site and report serious adverse events by phone to

Prof. Dr. Jennifer Keiser, Swiss Tropical and Public Health Institute, Tel.: +41 61 284-8218
Fax: +41 61 284-8105; E-mail: jennifer.keiser@unibas.ch

Within the following 48 hours, the co-investigator must provide further information on the serious adverse event or the unanticipated problem in the form of a written narrative. This should include a copy of the completed SAE form, and any other diagnostic information that will assist the understanding of the event. This information will be provided to ‘Ethikkomission Nordwest- und Zentralschweiz’ (EKNZ, Switzerland) and the comité national d’éthique et de la recherche (CNER, Côte d’Ivoire). Significant new information on ongoing serious adverse events should be provided promptly to the study sponsor.

7. Statistics

7.1. Definition of primary endpoint

CR and ERR is the primary endpoint in our study.

7.2. Justification of number of trial subjects

The antischistosomal effect of moxidectin and Synriam® has not yet been studied. By definition, pilot studies are conducted to serve as a starting point for further studies and are primarily intended to yield information about the feasibility and implementation possibilities of novel treatments. Thus, the choice of an adequate sample size for a pilot study is mainly based on practical constraints of the pilot trial rather than on statistical sample size calculations. Allowing for up to 20% drop-outs, we aimed for 30 children per treatment arm.

For the estimation of the prevalence of *S. mansoni* and *S. haematobium* infection in the school-aged children of CNRA (60%) and Moapé (80%) respectively. Previous conducted studies at nearby sites were considered. The two studies of Keiser et *al*. and Utzinger et al. reported an infection of 60-70%. However, we used a conservative estimation of 60% for our sample size determination and hence we are planning to enrol 200 children in each study to detect at least children infected with *S. mansoni*/*S. haematobium* [23,24].

7.3. Description of statistical methods

An available case analysis, which is sometimes erroneously referred to as an intention-to-treat analysis, will be performed, including all children with primary end point data. In addition, a per-protocol analysis and an intention-to-treat analysis with a worst case and a best case scenario will be conducted. CRs will be calculated as the percentage of egg-positive children at baseline who become egg-negative after treatment. EPG will be assessed by adding up the egg counts from the duplicate Kato-Katz thick smears and multiplying this number by a factor of six. The ERR will be calculated (ERR = (1-(mean at follow-up/mean at baseline))*100). Differences among CRs will be analysed by using crude logistic regressions and adjusted logistic regressions (adjustment for age, sex, school, and height).

Geometric and arithmetic mean egg counts will be calculated for the different treatment arms before and after treatment to assess the corresponding ERRs. Bootstrap resampling method with 2,000 replicates will be used to calculate 95% confidence intervals (CIs) for ERRs. Differences in ERRs will be determined under the assumption that non-overlapping CIs indicate statistical significance.

7.4. Description of data management

The investigators are responsible for an adequate data quality. Prior to the initiation of the study, a short investigator's meeting will be held with the investigators and their study coordinators and a member from Swiss TPH. This meeting will include a detailed discussion of the protocol, performance of study procedures, CRF completion, and specimen collection and diagnostic methods.

Screened patients will be listed in a confidential “subject screening log”. Enrolled patients will be listed in a confidential “subject enrolment log” and attributed a unique study number; this document will constitute the only source to decode the pseudonymised data and will only be accessible to the local principal investigator. All data that have been hand-entered in the database will be verified by a double-key entry procedure in a validated electronic data base system and error, range and consistency checks will be programmed. Any discrepancies will be reviewed against the hard copy CRF and corrected. Electronic data files will be stored on secured network drives with restricted access for study personnel only. Data analysis will be conducted with pseudonymised data and reporting of findings will be fully anonymized.

Essential infrastructure such as lockable cabinets for safe storage of hardcopy data will be made available. Network drives with restricted access for authorized personnel only and appropriate analysis software are available.

8. Duties of the investigator

8.1. Investigator's confirmation

This trial will be conducted in compliance with the protocol, International Conference on Harmonisation Good Clinical Practice E6 (ICH-GCP) and the current version of the Helsinki Declaration.

All protocol modifications must be documented in writing. A protocol amendment can be initiated by either the Sponsor or any Investigator. The Investigator will provide the reasons for the proposed amendment in writing and will discuss with the Sponsor and the Principal Investigator. Any protocol amendment must be approved and signed by the Sponsor and the Principal Investigator and must be submitted to the appropriate Independent Ethics Committee (IEC) for information and approval, in accordance with local requirements, and to regulatory agencies if required. Approval by IEC must be received before any changes can be implemented, except for changes necessary to eliminate an immediate hazard to trial subjects, or when the change involves only logistical or administrative aspects of the trial, e.g. change of telephone number(s).

8.2. Damage coverage

The study will be financed through a grant from the Geigy Foundation and the ERC. A general liability insurance of the Swiss TPH is in place (Winterthur Police Nr. 4746321). We are currently in contact with the National Insurance Corporation of Côte d'Ivoire for acquiring an insurance for the study participants (confirmation will be delivered).

8.3. Project management

The trial team will include the PI (Prof. Jennifer Keiser), a trial and data manager (Beatrice Barda, MD), a trial statistician (Dr. Jan Hattendorf), as well as laboratory technicians. Prof. Jennifer Keiser and Dr. Beatrice Barda will be responsible for staff management, communication with the collaborative group, recruitment monitoring, data management, safety reporting, analysis, report writing and dissemination of the trial results. Dr. Jean Coulibaly (Co-Pi) is responsible for supervision of the lab- and field technicians, staff management, recruitment monitoring, supply of the material, contact to the local authorities and participating schools.

The investigator team is responsible for ensuring that the protocol is strictly followed. The investigator should not make any changes without the agreement of the Principal Investigator and the Co-Investigators, except when necessary to eliminate an apparent immediate hazard or danger to a study participant. The investigator will work according to the protocol and GCP. The investigator may take any steps judged necessary to protect the safety of the participants, whether specified in the protocol or not. Any such step must be documented. During the treatment the records are maintained by the responsible medical doctor. All entries have to be made clearly readable with a pen. The investigator must be thoroughly familiar with the properties, effects and safety of the investigational pharmaceutical product.

9. Ethical considerations

9.1. Independent Ethics Committee (IEC)

The study will be submitted for approval by the institutional research commission of the Swiss TPH, Centre Suisse de Recherches Scientifiques and the University Félix Houphouët Boigny. Ethical clearance will be sought from the ethics committee of Switzerland (EKNZ) and Côte d'Ivoire (CNER). The study will be undertaken in accordance with the Declaration of Helsinki and good clinical practice (GCP).

9.2. Evaluation of the risk-benefit ratio

Moxidectin, Synriam® and Praziquantel have little adverse events [17-22,33]. All children enrolled in the study will benefit from a treatment against STHs and *S. mansoni* and *S. haematobium*. All participating children remaining positive for *A. lumbricoides,* hookworm, *T. trichiura, S. stercoralis,* schistosomes and malaria will be treated with albendazole and/or praziquantel, and/or ivermectin and malaria treatment according to the national guidelines.

9.3. Subject information and consent

Parents or legal guardians of eligible children will be asked to sign a written informed consent sheet. Children will be asked orally for assent. Community meetings will be conducted to explain the purpose and procedures of the study. Participation is voluntary and children have the right to withdraw from the study at any given point in time with no further obligations. Participation itself will not be awarded with compensation.

9.4. Subject Confidentiality

Confidentiality of information will be assured to the participants. The investigators have all been trained in GCPs. None of the investigators declare to have any conflicts of interest.
Subject's anonymity will be maintained. Subjects will be identified on the Case Report Forms by the Subject Number and Subject's Initials in addition to centre and study identification information. The investigators will keep a separate confidential enrolment log that matches identifying codes with the subjects' names and residencies.

9.5. Subjects requiring particular protection

This study will be carried out in school-aged children, since an infection with *S. mansoni* and *S. haematobium* occurs most often in children, hence this age group is at highest risk of infection. Our trial will pave the way for a safe and effective treatment of schistosomiasis in children.

10. Quality control and quality assurance

10.1. Monitoring and auditing

We will work with locally based clinical monitors. They will conduct site visits to the investigational facilities for the purpose of monitoring the study. The investigator will permit them access to study documentation and the clinical supplies dispensing and storage area. Monitoring observations and findings will be documented and communicated to appropriate study personnel and management. A corrective and preventative action plan will be requested and documented in response to any audit observations. No sponsor initiated audits are foreseen, but audits and inspections may be conducted by the local regulatory authorities or ethics committees. The Investigator agrees to allow inspectors from regulatory agencies to review records and is encouraged to assist the inspectors in their duties, if requested.

10.2. Access to data, handling of data and samples (data protection), archiving (place, duration) and destruction

Information about study subjects will be kept confidential and managed accordingly. A CRF will be completed for each subject enrolled into the clinical study. The investigators will review, approve and sign/date each completed CRF; the investigator-sponsor's signature serving as attestation of the investigator-sponsor's responsibility for ensuring that all clinical and laboratory data entered on the CRF are complete, accurate and authentic. The study CRF is the primary data collection instrument for the study. All data requested on the CRF must be recorded. All missing data must be explained. If a space on the CRF is left blank because the procedure was not done or the question was not asked "N/D" will be entered. If the item is not applicable to the individual case "N/A" will be written. All entries will be printed in black ink. All corrections must be initialed and dated.

All data on parasitology and questionnaires about adverse events and self-reported clinical signs and symptoms will be doubled entered into a database by two independent persons and cross-checked. Discrepancies between data entries will be corrected by consulting the hard copy.

The collected data will be stored at server of the Swiss Tropical and Public Health Institute and are encrypted with Secure Sockets Layer (SSL).

The results of the research study will be published, but subjects' names or identities will not be revealed. Records will remain confidential. To maintain confidentiality, the PI will keep records in locked cabinets and the results of tests will be coded to prevent association with participant's names. Data entered into the EXCEL data entry mask will be accessible only by

authorized personnel directly involved with the study and will be encoded. Subject-specific information may be provided to other appropriate medical personnel only with the subject's permission.

After the study has been completed all samples will be destroyed and research data and related material will be kept for a minimum of 10 years to enable understanding of what was done, how and why, which allow the work to be assessed retrospectively and repeated if necessary.

10.3. Data entered directly in the Case Report Form (CRF) – definition of source data

Source Data are the clinical findings and observations, laboratory data maintained at the study site. Source data are contained in source documents. Local authorities are allowed to access the source data.
Source Documents are the physician's subject records maintained at the study site. When applicable, information recorded on the CRF shall match the *Source Data* recorded on the *Source Documents*. All CRFs will be kept for at least 10 years.

10.4. Data and safety monitoring board (WHO) / data monitoring committee (EU/FDA)

In our study no DSMB will established, since we work with a well-known drug in a small sample size and using a single dose treatment. However, advisors will be informed regularly and the findings discussed.

11. Dissemination of results and publication

The final results of this study will be shared with the local laboratory staff and later published in a scientific journal and presented at scientific conferences. All results from this investigation are considered confidential and shall not be made available to any third part by any member of the investigating team before publication.

12. References

1. Murray, C.J., et al., *Disability-adjusted life years (DALYs) for 291 diseases and injuries in 21 regions, 1990-2010: a systematic analysis for the Global Burden of Disease Study 2010*. Lancet, 2012. 380:2197-223
2. Colley, D.G., et al., *Human schistosomiasis.* Lancet, 2014. 383:2253-64
3. World Health Organization, *Preventive chemotherapy in human helminthiasis: coordinated use of anthelminthic drugs in control interventions: a manual for health professionals and programme managers*. 2006, Geneva.
4. Utzinger, J., et al., *From innovation to application: Social-ecological context, diagnostics, drugs and integrated control of schistosomiasis.* Acta Trop, 2011. 120 Suppl 1: 121-37.
5. Melman, S.D., et al., *Reduced susceptibility to praziquantel among naturally occurring Kenyan isolates of Schistosoma mansoni*. PLoS Negl Trop Dis, 2009. 3(8):e504.
6. Keiser, J. and J. Utzinger, *Antimalarials in the treatment of schistosomiasis*. Curr Pharm Des, 2012. 18:3531-8.
7. Pedrique, B., et al., *The drug and vaccine landscape for neglected diseases (2000-11): a systematic assessment*. The Lancet Global Health, 2013.1:371 - e379.
8. White, N.J., *Qinghaosu (artemisinin): the price of success*. Science, 2008. 320:330-4.
9. Keiser, J. and J. Utzinger, *Artemisinins and synthetic trioxolanes in the treatment of helminth infections*. Curr Opin Infect Dis, 2007. 20:605-12.
10. N'Goran, E.K. et al., *Randomized, double-blind, placebo-controlled trial of oral artemether for the prevention of patent Schistosoma haematobium infections*. Am J Trop Med Hyg, 2003. 68:24-32.
11. Xiao, S.H., et al., *The in vitro and in vivo activities of synthetic trioxolanes on major human schistosome species*. Antimicrobial Agents Chemother, 2007. 51:1440-1445.
12. CY, Patil, et al., *Fixed Dose Combination of Arterolane and Piperaquine: A Newer Prospect in Antimalarial*. Therapy Ann Med Health Sci Res, 2014. 4:466–471.
13. http://www.a2s2.org/upload/5.ArtemisininConferences/1.2013Kenya/Presentations/Day2/2.SynriamRanbaxy.pdf
14. Tagawa, M, et al., *Prophylactic efficacy of milbemycin D against Dirofilaria immitis infection in dogs*. Nihon Juigaku Zasshi. 1985. 47:787-90.
15. Bowman, DD, et al., *Effects of milbemycin on adult Toxocara canis in dogs with experimentally induced infection*. Am J Vet Res, 1988. 49:1986-9.

16. Tagboto, SK and Townson S. *Onchocerca volvulus and O. lienalis: the microfilaricidal activity of moxidectin compared with that of ivermectin in vitro and in vivo*. Ann Trop Med Parasitol, 1996. 90:497-505.
17. Cotreau, MM, et al., *The antiparasitic moxidectin: safety, tolerability, and pharmacokinetics in humans*. J Clin Pharmacol. 2003.43:1108-15.
18. Awadzi, K, et al., *A randomized, single-ascending-dose, ivermectin-controlled, double-blind study of moxidectin in Onchocerca volvulus infection.* PLoS Negl Trop Dis, 2014. 8:e2953.
19. Cobb, R and Boeckh, A. *Moxidectin: a review of chemistry, pharmacokinetics and use in horses.* Parasit Vectors, 2009. 2:S5.
20. Forrester, SG, et al., *A glutamate-gated chloride channel subunit from Haemonchus contortus: expression in a mammalian cell line, ligand binding, and modulation of anthelmintic binding by glutamate*. Biochem Pharmacol, 2002.63:1061-8.
21. Korth-Bradley, JM, et al., *Relative Bioavailability of Liquid and Tablet Formulations of the Antiparasitic Moxidectin*. Clinical Pharmacology in Drug Development, 2012. 1: 32–37.
22. Korth-Bradley, JM, et al., *The effect of a high-fat breakfast on the pharmacokinetics of moxidectin in healthy male subjects: a randomized phase I trial*. Am J Trop Med Hyg, 2012. 86:122-5.
23. Attah, SK, et al., *Effect of a single dose of 8 mg moxidectin or 150 µg/kg ivermectin on intestinal helminths in participants of a clinical trial conducted in North-East DRC, Liberia and Ghana*. Am Soc Trop Med Hyg, abstract conference 2014.
24. Keiser, J, et al., *Polyparasitism with Schistosoma mansoni, geohelminths, and intestinal protozoa in rural Côte d'Ivoire*. J Parasitol, 2002. 88:461-6.
25. Utzinger, J, et al., *Schistosoma mansoni, intestinal parasites and perceived morbidity indicators in schoolchildren in a rural endemic area of western Côte d'Ivoire*. Trop Med Int Health, 1998. 9:711-20.
26. Pugh, RN., *A filtration method for schistosome egg quantification*. Ann Trop Med Parasitol, 1978. 72:387-8.
27. Katz, N., et al., *A simple device for quantitative stool thick-smear technique in Schistosomiasis mansoni*. Rev Inst Med Trop Sao Paulo, 1972. 14:397-400.
28. García, LS and Bruckner, DA., *Diagnostic Medical Parasitology.* Washington, DC: American Society for Microbiology, 2001.1–791.
29. Van Dam, GJ et al., *Schistosoma mansoni: in vitro and in vivo excretion of CAA and CCA by developing schistosomula and adult worms.* J Parasitol, 1996.82:557-64.

30. Keiser, J and Utzinger, J, *Efficacy of Current Drugs Against Soil-Transmitted Helminth Infections Systematic Review and Meta-analysis*. JAMA, 2008. 299:1937-1948.

31. Vercruysse, J et al., *Assessment of the anthelmintic efficacy of albendazole in school children in seven countries where soil-transmitted helminths are endemic*. PLoS Negl Trop Dis, 2011. 5:e948.

32. Reta, B and Erko, B. *Efficacy and side effects of praziquantel in the treatment for Schistosoma mansoni infection in school children in Senbete Town, northeastern Ethiopia*. Trop Med Int Health. 2013 18:1338-43.

Appendix 1: Kato-Katz manual from the World Health Organization (assessed: http://www.who.int/neglected_diseases/preventive_chemotherapy/pctnewsletter11.pdf; 05.03.2013).

KATO-KATZ KITS

Step 1: Label a glass slide[4] with the sample number and then place a plastic template on top of it.

Step 2: Place a small amount of the faecal sample on a newspaper and press a piece of nylon screen on top. Using a spatula, scrape the sieved faecal material through the screen so that only the debris remains.

Step 3: Scrape up some of the sieved faeces to fill the hole in the template, avoiding air bubbles and levelling the faeces off to remove any excess.

Step 4: Carefully lift off the template and place it in a bucket of water mixed with concentrated detergent so that it can be reused.

Steps 5 and 6 of the Kato-Katz technique

Step 5: Place one piece of the cellophane, which has been soaked overnight in methylene blue glycerol solution, over the faecal sample.

Step 6: Place a clean slide over the top and press it evenly downwards to spread the faeces in a circle. If done well, it should be possible to read newspaper print through the stool smear.

Steps 5 and 6 of the Sandwich technique

Step 5: Turn the slide containing the small amount of stool upside down and place it on a clean slide to make a "sandwich".

Step 6: Using a circular motion, press the top slide firmly onto the bottom slide to spread the stool in an even circular layer. If done well, it should be possible to read newspaper print through the stool smear.

IMPORTANT: A slide prepared using the Sandwich technique must be read within 1 hour.

Step 7: If hookworm is present in the area the slide should be read within 30–60 minutes, irrespective of the technique used. After that time, the hookworm eggs disappear.

Place the slide under a microscope and examine the whole area in a systematic zigzag pattern. If the sample created by the Sandwich technique is too thick, it may be necessary to use a x400 magnification objective. Record the number and the type of each egg on a recording form alongside the sample number. Finally, multiply the number of eggs by 24[5] to give the number of eggs per gram (epg) – the standard measurement to assess the intensity of infection.

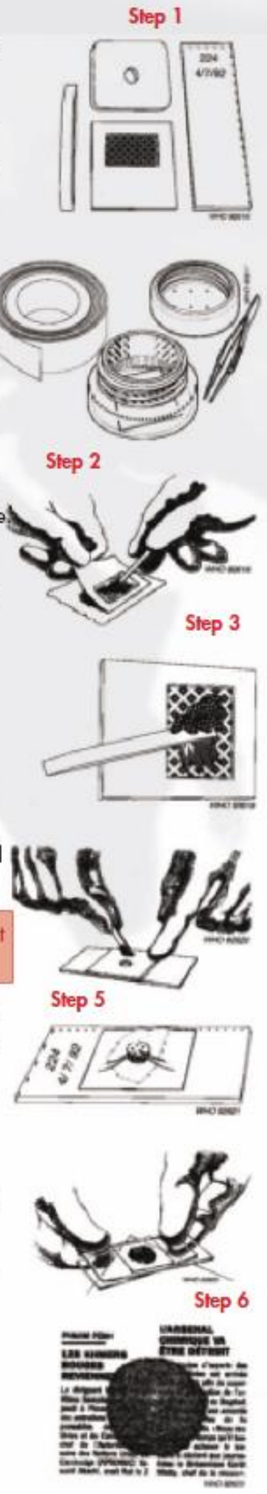

[4] The Kato-Katz technique uses a 25 x 75 mm slide. The Sandwich technique uses a 58 x 75 mm slide.

[5] This assumes that the standard 41.7 mg template is used. If a template of another size is used, multiply the number of eggs by the correct multiplier to give the epg.

SHORTAGES OF KATO-KATZ KITS
Sandwich or Teesdale technique

Kato-Katz kits contain a roll of cellophane that is cut into small pieces and soaked in methylene blue glycerol solution (not included in the kit) the night before the field work. The cellophane is then placed directly on the faeces sample, making the eggs more easily visible and allowing long-term storage of the slides.

Unfortunately, the company that supplied the bulk of the cellophane rolls recently discontinued production, resulting in a shortage of kits. WHO is therefore field-testing cellophane produced by alternative manufacturers to identify a high-quality product. Until the situation is resolved, the Sandwich or Teesdale technique, which is very similar to the Kato-Katz technique, is the most appropriate alternative. There is one important difference: the slides prepared with the Sandwich technique must be read within one hour of preparation.

[2] *Preventive chemotherapy in human helminthiasis: coordinated use of anthelminthic drugs in control interventions: a manual for health professionals and programme managers*. Geneva, World Health Organization, 2006 (available at: http://whqlibdoc.who.int/publications/2006/9241547103_eng.pdf).

[3] For example, the modified Ritchie technique.

Appendix 2: Urine filtration from the World Health Organization Manual in Basic Laboratory Methods in Human Parasitology

1. Place a polycarbonate or nylon filter (pore size 12–20 μm) in the filter holder. Alternatively, paper filters (Whatman No. 541 or No. 1) can be used. Agitate the urine sample by shaking it gently or by filling and emptying the syringe twice.
2. Draw 10 ml of the urine into the syringe and attach the filter-holder to the bottom of the syringe. (If less than 10 ml is available record in notebook.)
3. Keeping the unit level, expel the urine from the syringe into the filter holder over a bucket or sink.

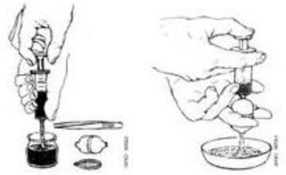

4. Carefully unscrew the filter holder, draw air into the syringe, reattach the syringe to the holder, and expel the air. (This is important as it helps to remove excess urine and also makes sure the eggs, if present, are attached to the filter.)
5. Unscrew the filter holder, remove the filter with the forceps and place it (top side up) on a microscope slide. Add one drop of Lugol's iodine and wait for 15 seconds for the stain to penetrate the eggs.

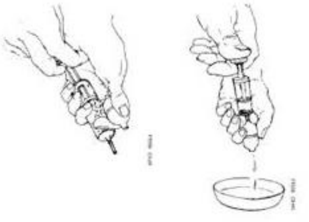

6. Examine the whole filter under the microscope immediately at low power (×40). Schistosome eggs stain orange and can be seen clearly. Infection loads are recorded as the number of eggs per 10 ml of urine. Therefore it is important to note the amount of urine examined, if it is less than 10 ml. To estimate the intensity of infection of the sample, divide the number of eggs counted by 10. If less than 10 ml was examined use the following equation:

$$\text{number of eggs per 10 ml sample} = \frac{\text{number of eggs counted}}{x} \times 10$$

where x = no. of ml of filtered urine examined.

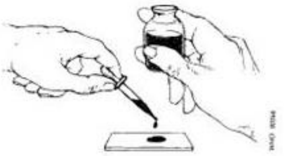

Appendix 3: Baermann technique

1. Set up ring stand. Attach hose to funnel and place funnel with hose into ring of ring stand. Secure clamp to hose attached to funnel. Place circular piece of wire screen inside of funnel.
2. Add distilled water to the funnel until the water surface is barely touching the wire. At this time, make sure that water is not leaking from the tube. If tube is leaking, re-adjust hose clamp until leaking stops.
3. Place an part of the faecal sample into a gauze and fold all four corners of the gauze.
4. Place the gauze into the wire, it should be dipped into the water.
5. The Baermann funnel should sit for approximately 2-4 hours to allow the nematodes to flow towards fresh water

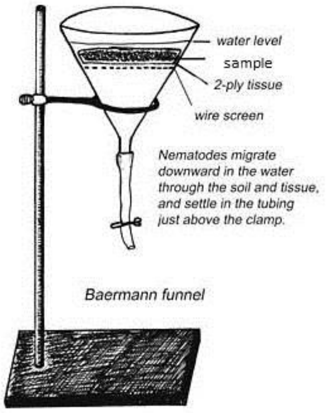

Supplement: S1 Protocol — (PDF) [file pntd.0005008.s002.pdf]
