# Supplementary material for: Efficacy and Safety of Moxidectin, Synriam, Synriam-Praziquantel versus Praziquantel against Schistosoma haematobium and S. mansoni Infections: A Randomized, Exploratory Phase 2 Trial
Source: PLoS Negl Trop Dis. 2016 Sep 16;10(9):e0005008. doi: 10.1371/journal.pntd.0005008 (PMC5026339; doi:10.1371/journal.pntd.0005008)
Supplement: S1 Table — (DOCX) [file pntd.0005008.s003.docx]

**Table S1: Number of children with clinical symptoms prior to treatment and adverse events among the four different treatment arms assessed at different time points in the *S. haematobium* cohort**

|  |  |  | **Moxidectin (N=31)** | **Synriam plus praziquantel (N=32)** | **Synriam (N=33)** | **Praziquantel (N=32)** |
| --- | --- | --- | --- | --- | --- | --- |
| Before treatment |  | Constipation | 15 (48.4) | 8 (25) | 10 (30.3) | 6 (18.8) |
|  |  | Headache | 9 (29) | 11 (34.4) | 16 (48.5) | 15 (46.9) |
|  |  | Dizziness | 1 (3.2) | 2 (6.3) | 6 (18.2) | 3 (9.4) |
|  |  | Stomach ache | 11 (35.5) | 11 (34.4) | 15 (45.5) | 11 (34.4) |
|  |  | Cough | 7 (22.6) | 13 (40.6) | 6 (18.2) | 11 (34.4) |
|  |  | Itching | 10 (32.3) | 7 (21.9) | 11 (33.3) | 10 (31.3) |
|  |  | Nausea | 4 (12.9) | 4 (12.5) | 6 (18.2) | 5 (15.6) |
|  |  | Vomiting | 2 (6.5) | 0 (0) | 3 (9.1) | 0 (0) |
|  |  | Diarrhea | 4 (12.9) | 6 (18.8) | 7 (21.2) | 6 (18.8) |
|  |  | Thrill | 0 (0) | 2 (6.3) | 3 (9.1) | 4 (12.5) |
|  |  | Fever | 9 (29) | 11 (34) | 13 (39.4) | 12 (37.5) |
| 1st day of treatment | 3h | Constipation | 3 (9.7) | 0 (0) | 0 (0) | 1 (3.1) |
|  |  | Headache | 4 (12.9) | 8 (25) | 5 (15.2) | 7 (21.9) |
|  |  | Dizziness | 1 (3.2) | 1 (3.1) | 1 (3) | 6 (18.8) |
|  |  | Stomach ache | 7 (22.6) | 7 (21.9) | 13 (39.4) | 11 (34.4) |
|  |  | Cough | 3 (9.7) | 0 (0) | 2 (6.1) | 4 (12.5) |
|  |  | Itching | 3 (9.7) | 0 (0) | 2 (6.1) | 3 (9.4) |
|  |  | Nausea | 4 (12.9) | 0 (0) | 9 (27.3) | 9 (28.1) |
|  |  | Vomiting | 0 (0) | 0 (0) | 1 (3) | 0 (0) |
|  |  | Diarrhea | 3 (9.7) | 0 (0) | 2 (6.1) | 1 (3.1) |
|  |  | Thrill | 0 (0) | 0 (0) | 1 (3) | 5 (15.6) |
|  |  | Fever | 0 (0) | 0 (0) | 0 (0) | 5 (15.6) |
|  | 24h | Constipation | 0 (0) | 1 (3.1) | 0 (0) | 1 (3.1) |
|  |  | Headache | 5 (16.1) | 3 (9.4) | 1 (3) | 2 (6.3) |
|  |  | Dizziness | 0 (0) | 0 (0) | 0 (0) | 0 (0) |
|  |  | Stomach ache | 5 (16.1) | 4 (12.5) | 4 (12.1) | 2 (6.3) |
|  |  | Cough | 0 (0) | 0 (0) | 0 (0) | 0 (0) |
|  |  | Itching | 2 (6.5) | 2 (6.3) | 0 (0) | 1 (3.1) |
|  |  | Nausea | 0 (0) | 0 (0) | 1 (3) | 0 (0) |
|  |  | Vomiting | 0 (0) | 0 (0) | 0 (0) | 0 (0) |
|  |  | Diarrhea | 0 (0) | 1 (3.1) | 0 (0) | 0 (0) |
|  |  | Thrill | 1 (3.2) | 0 (0) | 0 (0) | 2 (6.3) |
|  |  | Fever | 0 (0) | 0 (0) | 0 (0) | 0 (0) |
|  | 72h | Constipation | 0 (0) | 0 (0) | 0 (0) | 0 (0) |
|  |  | Headache | 5 (16.1) | 3 (9.4) | 1 (3) | 2 (6.3) |
|  |  | Dizziness | 1 (3.2) | 0 (0) | 0 (0) | 0 (0) |
|  |  | Stomach ache | 2 (6.5) | 5 (15.6) | 5 (15.2) | 2 (6.3) |
|  |  | Cough | 0 (0) | 0 (0) | 0 (0) | 0 (0) |
|  |  | Itching | 1 (3.2) | 1 (3.1) | 0 (0) | 1 (3.1) |
|  |  | Nausea | 1 (3.2) | 0 (0) | 0 (0) | 0 (0) |
|  |  | Vomiting | 0 (0) | 1 (3.1) | 0 (0) | 0 (0) |
|  |  | Diarrhea | 1 (3.2) | 0 (0) | 0 (0) | 1 (3.1) |
|  |  | Thrill | 1 (3.2) | 0 (0) | 0 (0) | 0 (0) |
|  |  | Fever | 1 (3.2) | 0 (0) | 0 (0) | 0 (0) |
| 2nd day of treatment | 3h | Constipation |  | 0 (0) | 0 (0) |  |
|  |  | Headache |  | 0 (0) | 0 (0) |  |
|  |  | Dizziness |  | 0 (0) | 0 (0) |  |
|  |  | Stomach ache |  | 2 (6.3) | 0 (0) |  |
|  |  | Cough |  | 0 (0) | 0 (0) |  |
|  |  | Itching |  | 0 (0) | 0 (0) |  |
|  |  | Nausea |  | 0 (0) | 2 (6.1) |  |
|  |  | Vomiting |  | 2 (6.3) | 0 (0) |  |
|  |  | Diarrhea |  | 0 (0) | 0 (0) |  |
|  |  | Thrill |  | 0 (0) | 0 (0) |  |
|  |  | Fever |  | 0 (0) | 0 (0) |  |
|  | 24h | Constipation |  | 0 (0) | 0 (0) |  |
|  |  | Headache |  | 0 (0) | 0 (0) |  |
|  |  | Dizziness |  | 0 (0) | 0 (0) |  |
|  |  | Stomach ache |  | 1 (3.1) | 0 (0) |  |
|  |  | Cough |  | 0 (0) | 0 (0) |  |
|  |  | Itching |  | 0 (0) | 0 (0) |  |
|  |  | Nausea |  | 0 (0) | 0 (0) |  |
|  |  | Vomiting |  | 0 (0) | 0 (0) |  |
|  |  | Diarrhea |  | 0 (0) | 0 (0) |  |
|  |  | Thrill |  | 0 (0) | 0 (0) |  |
|  |  | Fever |  | 0 (0) | 0 (0) |  |
|  | 72h | Constipation |  | 0 (0) | 0 (0) |  |
|  |  | Headache |  | 0 (0) | 0 (0) |  |
|  |  | Dizziness |  | 0 (0) | 0 (0) |  |
|  |  | Stomach ache |  | 0 (0) | 0 (0) |  |
|  |  | Cough |  | 0 (0) | 0 (0) |  |
|  |  | Itching |  | 0 (0) | 0 (0) |  |
|  |  | Nausea |  | 0 (0) | 0 (0) |  |
|  |  | Vomiting |  | 0 (0) | 0 (0) |  |
|  |  | Diarrhea |  | 0 (0) | 0 (0) |  |
|  |  | Thrill |  | 0 (0) | 0 (0) |  |
|  |  | Fever |  | 0 (0) | 0 (0) |  |
| 3rd day of treatment | 3h | Constipation |  | 0 (0) | 0 (0) |  |
|  |  | Headache |  | 0 (0) | 0 (0) |  |
|  |  | Dizziness |  | 0 (0) | 0 (0) |  |
|  |  | Stomach ache |  | 1 (3.1) | 0 (0) |  |
|  |  | Cough |  | 0 (0) | 0 (0) |  |
|  |  | Itching |  | 0 (0) | 0 (0) |  |
|  |  | Nausea |  | 0 (0) | 0 (0) |  |
|  |  | Vomiting |  | 0 (0) | 0 (0) |  |
|  |  | Diarrhea |  | 0 (0) | 0 (0) |  |
|  |  | Thrill |  | 0 (0) | 0 (0) |  |
|  |  | Fever |  | 0 (0) | 0 (0) |  |
|  | 24h | Constipation |  | 0 (0) | 0 (0) |  |
|  |  | Headache |  | 1 (3.1) | 0 (0) |  |
|  |  | Dizziness |  | 1 (3.1) | 0 (0) |  |
|  |  | Stomach ache |  | 0 (0) | 0 (0) |  |
|  |  | Cough |  | 0 (0) | 0 (0) |  |
|  |  | Itching |  | 0 (0) | 0 (0) |  |
|  |  | Nausea |  | 0 (0) | 0 (0) |  |
|  |  | Vomiting |  | 0 (0) | 0 (0) |  |
|  |  | Diarrhea |  | 0 (0) | 0 (0) |  |
|  |  | Thrill |  | 0 (0) | 0 (0) |  |
|  |  | Fever |  | 0 (0) | 0 (0) |  |
|  | 72h | Constipation |  | 0 (0) | 0 (0) |  |
|  |  | Headache |  | 0 (0) | 0 (0) |  |
|  |  | Dizziness |  | 0 (0) | 0 (0) |  |
|  |  | Stomach ache |  | 0 (0) | 0 (0) |  |
|  |  | Cough |  | 0 (0) | 0 (0) |  |
|  |  | Itching |  | 0 (0) | 0 (0) |  |
|  |  | Nausea |  | 0 (0) | 0 (0) |  |
|  |  | Vomiting |  | 0 (0) | 0 (0) |  |
|  |  | Diarrhea |  | 0 (0) | 0 (0) |  |
|  |  | Thrill |  | 0 (0) | 0 (0) |  |
|  |  | Fever |  | 0 (0) | 0 (0) |  |
