# Supplementary material for: Efficacy and Safety of Moxidectin, Synriam, Synriam-Praziquantel versus Praziquantel against Schistosoma haematobium and S. mansoni Infections: A Randomized, Exploratory Phase 2 Trial
Source: PLoS Negl Trop Dis. 2016 Sep 16;10(9):e0005008. doi: 10.1371/journal.pntd.0005008 (PMC5026339; doi:10.1371/journal.pntd.0005008)
Supplement: S2 Table — (DOCX) [file pntd.0005008.s004.docx]

**Table S2: Number of children with clinical symptoms prior to treatment and with adverse events among the four different treatment arms assessed at different time points in the *S. mansoni* cohort**

|  |  |  | **Moxidectin (N=34)** | **Synriam plus praziquantel (N=30)** | **Synriam (N=32)** | **Praziquantel (N=32)** |
| --- | --- | --- | --- | --- | --- | --- |
| Before treatment |  | Constipation | 1 (2.9) | 2 (6.7) | 1 (3.1) | 0 (0) |
|  |  | Headache | 20 (58.8) | 17 (56.7) | 18 (56.3) | 14 (43.8) |
|  |  | Dizziness | 1 (2.9) | 2 (6.7) | 2 (6.3) | 1 (3.1) |
|  |  | Stomach ache | 19 (55.9) | 12 (40) | 14 (43.8) | 11 (34.4) |
|  |  | Cough | 9 (26.5) | 11 (36.7) | 13 (40.6) | 14 (43.4) |
|  |  | Itching | 8 (23.5) | 5 (16.7) | 7 (21.9) | 9 (28.1) |
|  |  | Nausea | 3 (8.8) | 3 (10) | 6 (18.8) | 4 (12.5) |
|  |  | Vomiting | 5 (14.7) | 6 (20) | 6 (18.8) | 4 (12.5) |
|  |  | Diarrhea | 12 (35.3) | 5 (16.7) | 10 (31.3) | 7 (21.9) |
|  |  | Thrill | 4 (11.8) | 7 (23.3) | 7 (21.9) | 7 (21.9) |
|  |  | Fever | 3 (8.8) | 2 (6.7) | 1 (3.1) | 1 (3.1) |
| 1st day of treatment | 3h | Constipation | 0 (0) | 0 (0) | 0 (0) | 0 (0) |
|  |  | Headache | 4 (11.8) | 5 (14.7) | 3 (9.4) | 1 (3.1) |
|  |  | Dizziness | 1 (2.9) | 1 (3.3) | 4 (12.5) | 1 (3.1) |
|  |  | Stomach ache | 8 (23.5) | 13 (43.3) | 6 (18.8) | 10 (31.3) |
|  |  | Cough | 5 (14.7) | 3 (10) | 1 (3.1) | 2 (6.3) |
|  |  | Itching | 3 (8.8) | 2 (6.7) | 1 (3.1) | 3 (9.38) |
|  |  | Nausea | 3 (8.8) | 3 (10) | 3 (9.4) | 3 (9.4) |
|  |  | Vomiting | 0 (0) | 5 (16.7) | 2 (6.3) | 3 (9.4) |
|  |  | Diarrhea | 2 (5.9) | 3 (10) | 0 (0) | 6 (18.8) |
|  |  | Thrill | 0 (0) | 3 (10) | 2 (6.3) | 1 (3.1) |
|  |  | Fever | 2 (5.9) | 3 (10) | 1 (3.1) | 3 (9.4) |
|  | 24h | Constipation | 0 (0) | 0 (0) | 1 (3.1) | 0 (0) |
|  |  | Headache | 2 (5.9) | 1 (3.3) | 1 (3.1) | 3 (9.4) |
|  |  | Dizziness | 0 (0) | 1 (3.3) | 0 (0) | 0 (0) |
|  |  | Stomach ache | 3 (8.8) | 5 (16.7) | 0 (0) | 2 (6.3) |
|  |  | Cough | 3 (8.8) | 4 (13.3) | 3 (9.4) | 3 (9.4) |
|  |  | Itching | 1 (2.9) | 6 (20) | 0 (0) | 3 (9.4) |
|  |  | Nausea | 0 (0) | 1 (3.3) | 0 (0) | 0 (0) |
|  |  | Vomiting | 0 (0) | 0 (0) | 0 (0) | 0 (0) |
|  |  | Diarrhea | 3 (8.8) | 1 (3.3) | 1 (3.1) | 1 (3.1) |
|  |  | Thrill | 0 (0) | 2 (6.7) | 0 (0) | 2 (6.3) |
|  |  | Fever | 1 (2.9) | 2 (6.7) | 0 (0) | 0 (0) |
|  | 72h | Constipation | 0 (0) | 0 (0) | 0 (0) | 0 (0) |
|  |  | Headache | 2 (5.9) | 0 (0) | 2 (6.3) | 2 (6.3) |
|  |  | Dizziness | 1 (2.9) | 0 (0) | 0 (0) | 0 (0) |
|  |  | Stomach ache | 3 (8.8) | 4 (13.3) | 1 (3.1) | 2 (6.3) |
|  |  | Cough | 0 (0) | 0 (0) | 0 (0) | 0 (0) |
|  |  | Itching | 0 (0) | 0 (0) | 0 (0) | 0 (0) |
|  |  | Nausea | 0 (0) | 1 (3.3) | 0 (0) | 0 (0) |
|  |  | Vomiting | 0 (0) | 1 (3.3) | 0 (0) | 0 (0) |
|  |  | Diarrhea | 2 (5.9) | 1 (3.3) | 1 (3.1) | 1 (3.1) |
|  |  | Thrill | 2 (5.9) | 0 (0) | 0 (0) | 0 (0) |
|  |  | Fever | 1 (2.9) | 0 (0) | 0 (0) | 0 (0) |
| 2nd day of treatment | 3 h | Constipation |  | 0 (0) | 0 (0) |  |
|  |  | Headache |  | 0 (0) | 0 (0) |  |
|  |  | Dizziness |  | 0 (0) | 0 (0) |  |
|  |  | Stomach ache |  | 0 (0) | 0 (0) |  |
|  |  | Cough |  | 0 (0) | 0 (0) |  |
|  |  | Itching |  | 0 (0) | 0 (0) |  |
|  |  | Nausea |  | 0 (0) | 0 (0) |  |
|  |  | Vomiting |  | 1 (3.3) | 0 (0) |  |
|  |  | Diarrhea |  | 0 (0) | 0 (0) |  |
|  |  | Thrill |  | 0 (0) | 0 (0) |  |
|  |  | Fever |  | 0 (0) | 0 (0) |  |
|  | 24h | Constipation |  | 0 (0) | 0 (0) |  |
|  |  | Headache |  | 0 (0) | 0 (0) |  |
|  |  | Dizziness |  | 0 (0) | 0 (0) |  |
|  |  | Stomach ache |  | 0 (0) | 0 (0) |  |
|  |  | Cough |  | 0 (0) | 0 (0) |  |
|  |  | Itching |  | 1 (3.3) | 0 (0) |  |
|  |  | Nausea |  | 0 (0) | 0 (0) |  |
|  |  | Vomiting |  | 0 (0) | 0 (0) |  |
|  |  | Diarrhea |  | 0 (0) | 0 (0) |  |
|  |  | Thrill |  | 0 (0) | 0 (0) |  |
|  |  | Fever |  | 0 (0) | 0 (0) |  |
|  | 72h | Constipation |  | 0 (0) | 0 (0) |  |
|  |  | Headache |  | 0 (0) | 0 (0) |  |
|  |  | Dizziness |  | 0 (0) | 0 (0) |  |
|  |  | Stomach ache |  | 0 (0) | 0 (0) |  |
|  |  | Cough |  | 0 (0) | 0 (0) |  |
|  |  | Itching |  | 1 (3.3) | 0 (0) |  |
|  |  | Nausea |  | 0 (0) | 0 (0) |  |
|  |  | Vomiting |  | 0 (0) | 0 (0) |  |
|  |  | Diarrhea |  | 0 (0) | 0 (0) |  |
|  |  | Thrill |  | 0 (0) | 0 (0) |  |
|  |  | Fever |  | 0 (0) | 0 (0) |  |
| 3rd day of treatment | 3 hr | Constipation |  | 0 (0) | 0 (0) |  |
|  |  | Headache |  | 0 (0) | 0 (0) |  |
|  |  | Dizziness |  | 0 (0) | 0 (0) |  |
|  |  | Stomach ache |  | 0 (0) | 0 (0) |  |
|  |  | Cough |  | 0 (0) | 0 (0) |  |
|  |  | Itching |  | 1(3.3) | 0 (0) |  |
|  |  | Nausea |  | 0 (0) | 0 (0) |  |
|  |  | Vomiting |  | 0 (0) | 0 (0) |  |
|  |  | Diarrhea |  | 0 (0) | 0 (0) |  |
|  |  | Thrill |  | 0 (0) | 0 (0) |  |
|  |  | Fever |  | 0 (0) | 0 (0) |  |
|  | 24h | Constipation |  | 0 (0) | 0 (0) |  |
|  |  | Headache |  | 0 (0) | 1 (3.1) |  |
|  |  | Dizziness |  | 0 (0) | 0 (0) |  |
|  |  | Stomach ache |  | 0 (0) | 0 (0) |  |
|  |  | Cough |  | 0 (0) | 0 (0) |  |
|  |  | Itching |  | 1 (3.3) | 1 (3.1) |  |
|  |  | Nausea |  | 0 (0) | 0 (0) |  |
|  |  | Vomiting |  | 0 (0) | 0 (0) |  |
|  |  | Diarrhea |  | 0 (0) | 0 (0) |  |
|  |  | Thrill |  | 0 (0) | 0 (0) |  |
|  |  | Fever |  | 0 (0) | 0 (0) |  |
|  | 72h | Constipation |  | 0 (0) | 0 (0) |  |
|  |  | Headache |  | 0 (0) | 0 (0) |  |
|  |  | Dizziness |  | 0 (0) | 0 (0) |  |
|  |  | Stomach ache |  | 0 (0) | 0 (0) |  |
|  |  | Cough |  | 0 (0) | 0 (0) |  |
|  |  | Itching |  | 1 (3.3) | 0 (0) |  |
|  |  | Nausea |  | 0 (0) | 0 (0) |  |
|  |  | Vomiting |  | 0 (0) | 0 (0) |  |
|  |  | Diarrhea |  | 0 (0) | 0 (0) |  |
|  |  | Thrill |  | 0 (0) | 0 (0) |  |
|  |  | Fever |  | 0 (0) | 0 (0) |  |
